# Integrative epigenome-wide analysis demonstrates that DNA methylation may mediate genetic risk in inflammatory bowel disease

**DOI:** 10.1038/ncomms13507

**Published:** 2016-11-25

**Authors:** N. T. Ventham, N. A. Kennedy, A. T. Adams, R. Kalla, S. Heath, K. R. O'Leary, H. Drummond, IBD BIOM consortium, Gordan Lauc, Harry Campbell, Dermot P. B. McGovern, Vito Annese, Vlatka Zoldoš, Iain K. Permberton, Manfred Wuhrer, Daniel Kolarich, Daryl L. Fernandes, Evropi Theorodorou, Victoria Merrick, Daniel I. Spencer, Richard A. Gardner, Ray Doran, Archana Shubhakar, Ray Boyapati, Igor Rudan, Paolo Lionetti, Irena Trbojević Akmačić, Jasminka Krištić, Frano Vučković, Jerko Štambuk, Mislav Novokmet, Maja Pučić-Baković, Olga Gornik, Angelo Andriulli, Laura Cantoro, Giancarlo Sturniolo, Gionata Fiorino, Natalia Manetti, Anna Latiano, Anna Kohn, Renata D'Incà, Silvio Danese, Ian D. Arnott, Colin L. Noble, Charlie W. Lees, Alan G. Shand, Gwo-Tzer Ho, Malcolm G. Dunlop, Lee Murphy, Jude Gibson, Louise Evenden, Nicola Wrobel, Tamara Gilchrist, Angie Fawkes, Guinevere S. M. Kammeijer, Florent Clerc, Noortje de Haan, Aleksandar Vojta, Ivana Samaržija, Dora Markulin, Marija Klasić, Paula Dobrinić, Yurii Aulchenko, Tim van den Heuve, Daisy Jonkers, Marieke Pierik, IBD CHARACTER consortium, Simen Vatn, Petr Ricanek, Jørgen Jahnsen, Panpan You, Janne Sølvernes, Anna B. Frengen, Tone M. Tannæs, Aina E. F. Moen, Fredrik A. Dahl, Jonas Christoffer Lindstrøm, Gunn S. Ekeland, Trond Espen Detlie, Åsa V. Keita, Johan D. Söderholm, Henrik Hjortswang, Jonas Halfvarson, Daniel Bergemalm, Fernando Gomollón, Mauro D'Amato, Leif Törkvist, Fredrik Hjelm, Mats Gullberg, Niklas Nordberg, Anette Ocklind, Erik Pettersson, Daniel Ekman, Mikael Sundell, Eddie Modig, Anne- Clémence Veillard, Renaud Schoemans, Dominique Poncelet, Céline Sabatel, Marta Gut, Monica Bayes, Christina Casén, Torbjørn Lindahl, Ewa Ciemniejewska, Morten H. Vatn, D. C. Wilson, I. G. Gut, E. R. Nimmo, J. Satsangi

**Affiliations:** 1 Gastrointestinal Unit, Centre for Genomics and Molecular Medicine, University of Edinburgh, Edinburgh EH4 6XU, UK; 2 CNAG-CRG, Centro Nacional de Análisis Genómico, Centre for Genomic Regulation (CRG), Barcelona Institute of Science and Technology (BIST), Baldiri i Reixac 4, Barcelona 08028, Spain; 3 Universitat Pompeu Fabra (UPF), Barcelona 08002, Spain; 4 Department of Child Life and Health, University of Edinburgh, Edinburgh EH9 1UW, UK; 5 Genos Glycoscience Research Laboratory, Hondlova 2/11, Zagreb 10000, Croatia; 6 Centre for Population Health Sciences, University of Edinburgh, Edinburgh EH8 9AG, UK; 7 F.Widjaja Family Foundation Inflammatory Bowel and Immunobiology Research Institute, Cedars-Sinai Medical Center, Los Angeles, California 90048, USA; 8 Department of Medical and Surgical Sciences, Division of Gastroenterology University Hospital Careggi, Largo Brambilla 3, Florence 50141, Italy; 9 Department of Biochemistry and Molecular Biology, University of Zagreb Faculty of Pharmacy and Biochemistry, A. Kovačića 1, Zagreb HR-10000, Croatia; 10 IP Research Consulting SAS, 34 rue Carnot 93160 Noisy-le-grand, Paris, France; 11 Center for Proteomics and Metabolomics, Leiden University Medical Center, Zone S3 Albinusdreef 2, Leiden 2333 ZA, The Netherlands; 12 Division of BioAnalytical Chemistry, VU University Amsterdam, De Boelelaan 1083, room M-352, Amsterdam 1081 HV, The Netherlands; 13 Department of Biomolecular Systems, Max Planck Institute of Colloids and Interfaces, Potsdam-Golm Science Park, Potsdam D-14424, Germany; 14 Ludger Ltd, Culham Science Centre, Oxford OX14 3EB, UK; 15 Paediatric Gastroenterology Unit, AOU Meyer, Viale Pieraccini, Viale Pieraccini 24, Florence 50139, Italy; 16 Department of Medical Sciences, Division of Gastroenterology, IRCCS-CSS Hospital, Viale Cappuccini, 1, S. Giovanni Rotondo, Rotondo, Italy; 17 Division of Gastroenterology, S. Camillo Hospital, Via Portuense 332, Rome I-00149, Italy; 18 Gastrointestinal Unit, University of Padua, Hospital Via Giustiniani 2, Padua 35128, Italy; 19 IBD Unit, Humanitas Research Institute, Rozzano, Via Manzoni 56, Milan 20089, Italy; 20 Department of Gastroenterology, Western General Hospital, Edinburgh EH4 6XU, UK; 21 Colon Cancer Genetics Group, Institute of Genetics and Molecular Medicine, University of Edinburgh and Medical Research Council Human Genetics Unit, Edinburgh EH4 6XU, UK; 22 Wellcome Trust Clinical Research Facility, University of Edinburgh, Western General Hospital, Edinburgh EH4 6XU, UK; 23 Institute of Cytology and Genetics SB RAS, Novosibirsk 630090, Russia; 24 Novosibirsk State University, Novosibirsk 630090, Russia; 25 Maastricht University Medical Centre (MUMC), P. Debyelaan 25, Maastricht 6229 HX, The Netherlands; 26 Akershus University Hospital, Department of Gastroenterology, Sykehusveien 25, Lørenskog 1478, Akershus, Norway; 27 Oslo University Hospital, Department of Gastroenterology, Bygg 6, 3 etg. Kirkeveien 166, Oslo N-0424, Norway; 28 Linköping University Hospital, Department of Surgery, Linköping 581 83, Sweden; 29 Department of Gastroenterology and Hepatology, UHL, County Council of Östergötland, Linköping 581 85, Sweden; 30 Örebro University, Department of Gastroenterology, Faculty of Medicine and Health, Långhuset, Fakultetsgatan 1, Örebro 702 81, Sweden; 31 University of Zaragoza, Gastroenterology, Calle de Pedro Cerbuna, 12, Zaragoza 50009, Spain; 32 BioCruces Health Research Institute, Cruces Plaza, Barakaldo, Bilbao 48903, Spain; 33 Department of Biosciences and Nutrition, Karolinska Institutet, Stockholm SE-171 77, Sweden; 34 Olink Proteomics, Uppsala Science Park, Hammarskjölds väg 52A, Uppsala 752 37, Sweden; 35 Diagenode, Liege Science Park, Rue du Bois Saint-Jean 3, Liège 4102, Belgium; 36 Genetic Analysis, Storo, Nycoveien 2, Oslo 0485, Norway

## Abstract

Epigenetic alterations may provide important insights into gene-environment interaction in inflammatory bowel disease (IBD). Here we observe epigenome-wide DNA methylation differences in 240 newly-diagnosed IBD cases and 190 controls. These include 439 differentially methylated positions (DMPs) and 5 differentially methylated regions (DMRs), which we study in detail using whole genome bisulphite sequencing. We replicate the top DMP (*RPS6KA2*) and DMRs (*VMP1, ITGB2* and *TXK*) in an independent cohort. Using paired genetic and epigenetic data, we delineate methylation quantitative trait loci; *VMP1/microRNA-21* methylation associates with two polymorphisms in linkage disequilibrium with a known IBD susceptibility variant. Separated cell data shows that IBD-associated hypermethylation within the *TXK* promoter region negatively correlates with gene expression in whole-blood and CD8^+^ T cells, but not other cell types. Thus, site-specific DNA methylation changes in IBD relate to underlying genotype and associate with cell-specific alteration in gene expression.

## Abstract (web summary)

Epigenetic perturbations may be an important factor in diseases where both genes and environment play a role. Here, Ventham and colleagues show that DNA methylation changes in inflammatory bowel disease are related to the underlying genotype, and are associated with cell-specific changes to gene expression.

## Graphical abstract

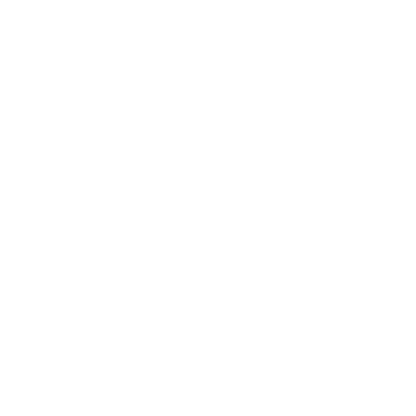

Inflammatory bowel disease (IBD) has a strong genetic contribution; a trans-ancestry meta-analysis of genome-wide associated studies (GWAS) has demonstrated 200 loci associated with IBD^1^. Despite this tremendous progress in delineating the genetic architecture of IBD, genetics explains only a small proportion of disease heritability (13.1% Crohn's disease (CD) and 8.2% ulcerative colitis (UC) of disease variance)^1^. Several environmental factors are known to influence the development and course of disease; particularly smoking, diet and the gut microbiota^2^. This has led some investigators to investigate epigenetics as a potential interface between genetics, environmental modifiers and disease^3^. Epigenome-wide association studies (EWAS) have provided insights into other complex diseases such as rheumatoid arthritis^4^, type 2 diabetes mellitus^5^ and obesity^6^.

DNA methylation EWAS aim to determine the distribution of methyl groups at thousands of specific positions across the genome (CpG sites, cytosine-guanine dinucleotide) with the aim of identifying arrangements that are more common to certain disease traits compared to controls^7^. The biological significance of DNA methylation is the association of DNA hypo- or hyper-methylation occurring within regulatory regions of genes (for example, promoters or transcription start sites) and gene repression^8^. Epigenetic studies have important confounding factors, most significantly, the cell-specific nature of epigenetic signatures^9^.

In the context of IBD several preliminary studies have used the Illumina 27k platform^10^,^11^,^12^ and subsequently the HumanMethylation450 platform to assess genome-wide DNA methylation patterns in blood and mucosal biopsy material in IBD^13^,^14^,^15^. In our own study in treatment-naive children with CD, highly significant differences in DNA methylation were noted to occur in genes implicated in disease pathogenesis^16^. The findings were replicable in a modest number of samples and a two DNA methylation probe biomarker was found to accurately discriminate IBD cases and controls, indicating a strong translational potential^16^.

In the present study, we use a comprehensive integrative approach to study multilevel DNA methylation, genomic and gene expression data and to relate changes seen in whole blood to the methylation profile in separated cells. In the primary analysis, we use the Illumina 450 K platform to identify differentially methylated positions (DMPs) and regions (DMRs) in whole-blood DNA samples from 240 newly diagnosed IBD cases (121 CD and 119 UC) and 191 controls. Technical validation and detailed characterization of DMRs is performed in a small cohort of 6 cases (3 CD and 3 UC) and 3 controls using whole-genome bisulphite sequencing. Independent validation of methylation results is performed using bisulphite pyrosequencing in a further cohort of 240 patients with established IBD and 98 controls. Differentially methylated sites discovered in whole blood are investigated in immunomagnetically separated leucocytes (CD4^+^ and CD8^+^ lymphocytes, CD14^+^ monocytes) in a subset of the main cohort (*n*=60). All patients and controls profiled using the Illumina 450K platform (*n*=431) are genotyped using the Illumina CoreExome-24 array that includes 547,644 genetic variants. In a subset of patients with separated cell data (*n*=68), we perform gene expression analysis using the Illumina HT12 microarray.

## Results

### IBD-associated site-specific differences in DNA methylation

**Figure 1:**
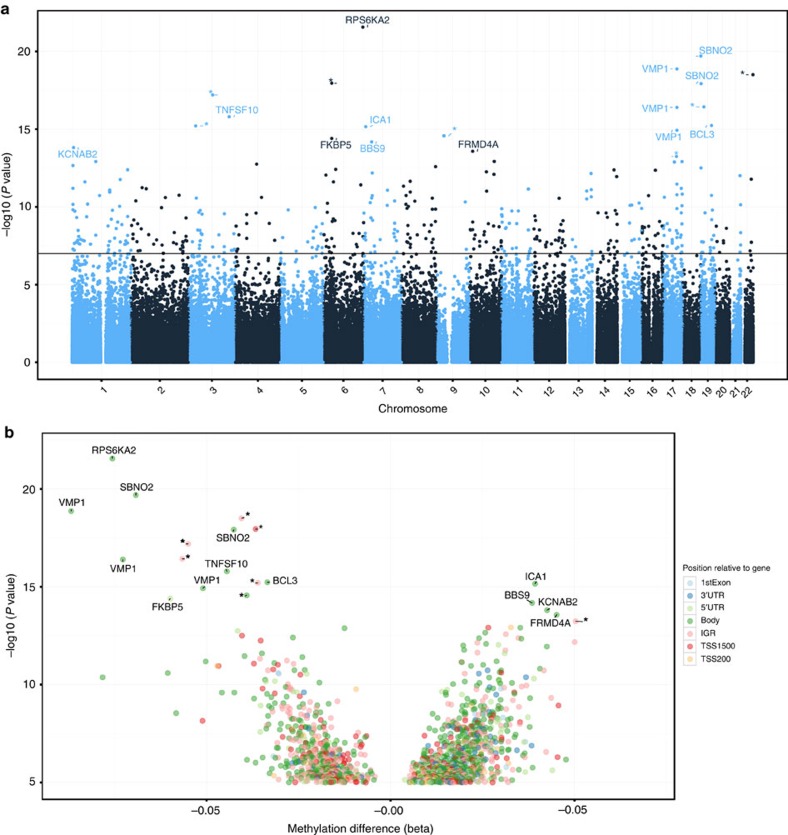
Differentially methylated positions (DMP) analysis in Inflammatory bowel disease (IBD) cases and controls in whole blood. (**a**) Manhattan plot of top differentially methylated positions (DMPs) in Inflammatory bowel disease (IBD) versus control. (**b**) Volcano plot of top DMPs and position of methylation probes in relation to the gene (IGR, intergenic region; TSS, transcription start site; UTR, untranslated region).

An epigenome-wide association comparison was made between IBD cases (both CD and UC) and controls (symptomatic and healthy controls, Supplementary Table 1). Linear models including age, sex and estimated cell proportions^17^ as covariates were used to estimate DMPs, with Holm adjustment^18^ used to stringently correct for multiple testing. The estimated cell proportions derived from the Houseman cell mixture deconvolution are presented in Supplementary Fig. 1. There were 439 DMPs in IBD cases compared with controls achieving corrected *P*<0.05 (Table 1, Fig. 1a,b). Gene ontology (GO) analysis revealed 54 significantly enriched GO terms, a large proportion of which relate to immune function (Supplementary Table 2). There were 412 DMPs when comparing CD to controls (Supplementary Table 3) and 203 when comparing UC to controls (Supplementary Table 4). As has previously been reported^15^, no significant differentially methylated sites were detected in a comparison of CD and UC following correction for multiple testing (Supplementary Table 5). There was a significant overlap between DMPs seen in IBD, CD and UC compared with controls (Supplementary Fig. 2). This parallels the latest genomic data where susceptibility loci initially thought to be associated with CD or UC are now known to be shared between both diseases^19^. There were no DMPs when comparing symptomatic controls and healthy volunteers (Supplementary Table 6), allowing grouping of symptomatic and healthy controls into a single large cohort.

**Figure 2:**
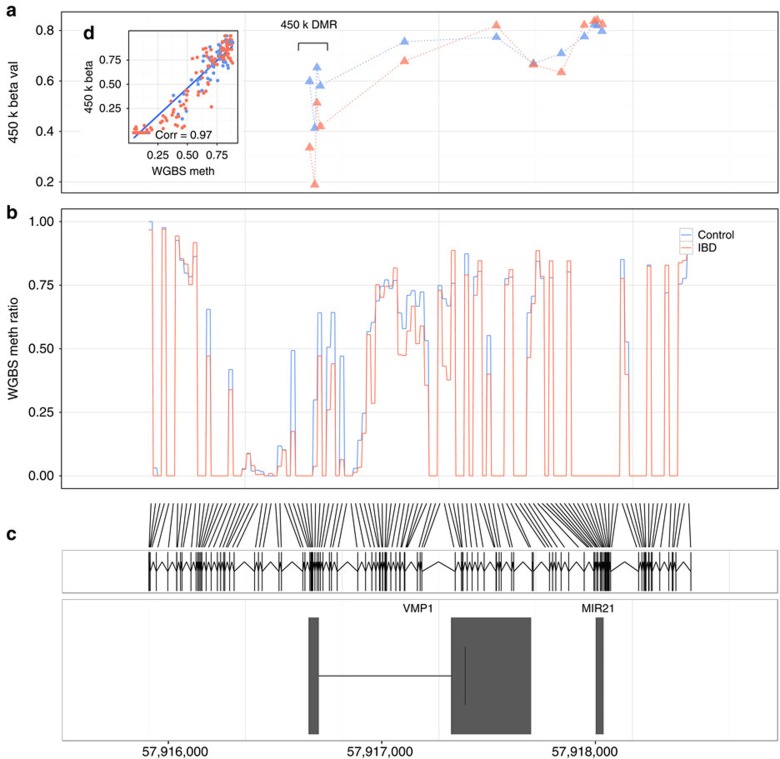
Detailed characterization of the VMP1/microRNA-21 region. (**a**) VMP1/microRNA-21 region 450 K microarray probes (triangle) in inflammatory bowel disease cases (IBD, Red) and controls (Blue). (**b**) the same VMP1/microRNA-21 region mapped using whole genome bisulphite sequencing (WGBS) data (Red line=IBD cases, Blue line=Controls). (**c**) VMP1/microRNA-21 gene schematic diagram. Note only the first two exons of VMP1 shown. (**d**) Correlation between 450k microarray probes and WGBS data at same site. Correlation using Pearson's test. X axis denotes Chr 17 (h19) coordinates. DMR, differentially methylated region in IBD versus control case control 450k analysis.

Differentially methylated regions (DMRs), the most compelling method of analysing DNA methylation data, were defined as ≥3 contiguous probes within a 2 kb distance with unidirectional methylation change and attaining Holm-adjusted *P*<0.05 on DMP linear model analysis. Five DMRs were identified in IBD cases versus controls and are listed in Table 2. There were four CD-associated DMRs (*VMP1*, *ITGB2*, *WDR8* and *CDC42BPB*) and two UC-associated DMRs (*VMP1* and *WDR8*) compared with controls. These DMRs have been studied in detail using whole genome bisulphite sequencing (Fig. 2) and are compelling biological targets for further investigation (Supplementary Table 7).

### Environmental modifiers of DNA methylation

The most significant DMP and DMRs were investigated for a possible association with inflammation. There was a strong correlation in both cases and controls between the top DMP and the inflammatory marker C-reactive protein (CRP and *RPS6KA2*, Spearman's Rho −0.53, *P*=1.8 × 10^−13^, Supplementary Fig. 3A), albumin (Pearson's correlation=0.94, *P*<2.2 × 10^−16^, Supplementary Fig. 3B) and haemoglobin (Pearson's correlation=0.55, *P*=6.7 × 10^−7^, Supplementary Fig. 3C). The association between CRP and RPS6KA2 remained consistent when outliers were removed (Supplementary Fig. 4). Moreover, hypomethylation of *RPS6KA2* was associated with a more severe maximum Montreal disease behaviour in CD (Kruskall–Wallis, *P*=0.047, Supplementary Fig. 3E) and more extensive disease in UC (Kruskall–Wallis, *P*=7.9 × 10^−5^, Supplementary Fig. 3F). A similar effect was seen with the top DMR (*VMP1*).

The potential impact of immunomodulatory therapy on the epigenetic profile has been studied in a subset of patients with data on treatment status at the time of blood sampling (139 IBD cases (64 treatment naive), 191 controls). Details on the specific therapies received are listed in Supplementary Table 1. The treatment status did not alter DNA methylation at key DMRs (Supplementary Fig. 5) and DMPs (Supplementary Fig. 6) identified in preliminary analyses.

*Post hoc* analyses were performed to investigate the known effect of age and smoking on DNA methylation. A linear model was used to compare smokers and non-smokers amongst all subjects with IBD status and cell proportions as covariates. The methylation change (Δβ) in previously published smoking- associated methylation probes demonstrated significant correlation to the same probes in the present data set (Pearson's correlation=0.93, *P*=1.9 × 10^−10^ Supplementary Fig. 7A)^20^. Sensitivity analyses including smoking (Supplementary Table 8) and treatment status (Supplementary Table 9) as covariates linear models for IBD versus control DMP analyses did not significantly change the findings described above.

The ‘epigenetic age' of the samples was calculated using a modified version of the method described by Horvath and correlated highly with the actual age (Pearson's correlation=0.93, 95% confidence interval 0.91–0.94, Supplementary Fig. 7B)^21^. There was no difference in the calculated age acceleration between IBD cases and controls (Supplementary Fig. 7C).

### IBD-associated DNA methylation changes are highly replicable

A major hurdle of EWAS has been a lack of replicable findings, largely as a result of small sample sizes and confounding cellular heterogeneity. Pyrosequencing of whole blood bisulphite converted DNA of a subset of the main cohort (130 IBD cases and 101 controls,Supplementary Table 10) provided technical validation of the Illumina 450K array with strong correlation of methylation differences across platforms (Supplementary Table 11, Supplementary Fig. 8). While the Illumina 450K array is a mature platform and technical replication is no longer required, replication of findings in independent cohorts remains critical. Selected 450K microarray findings were replicated using pyrosequencing in an independent cohort (240 established IBD cases, 98 controls, Supplementary Table 12) with the same direction of methylation change for the most significant DMP (*RPS6KA2*, IBD versus controls *P*=1 × 10^−9^, Wilcoxon Rank Sum,) and DMRs (*VMP1 P*=1 × 10^−6^, *IGTB2 P*=2 × 10^−7^ and *TXK P*=4 × 10^−10^, Wilcoxon Rank Sum, Supplementary Fig. 9A).

Our previously published early-onset CD whole-blood methylation data provided an additional independent validation cohort (36 paediatric CD, 36 controls)^16^. There was a highly significant correlation between the difference in beta values for the top 5,000 DMPs between CD cases and controls in the present adult data set and the paediatric data set (Pearson's correlation=0.77, 95% confidence interval 0.76–0.78, *P* value <2.2 × 10^16^, Supplementary Fig. 9B) and many of the most significant DMPs were shared (Supplementary Table 13).

### Cell-type specificity of whole tissue DNA methylation signals

**Figure 3:**
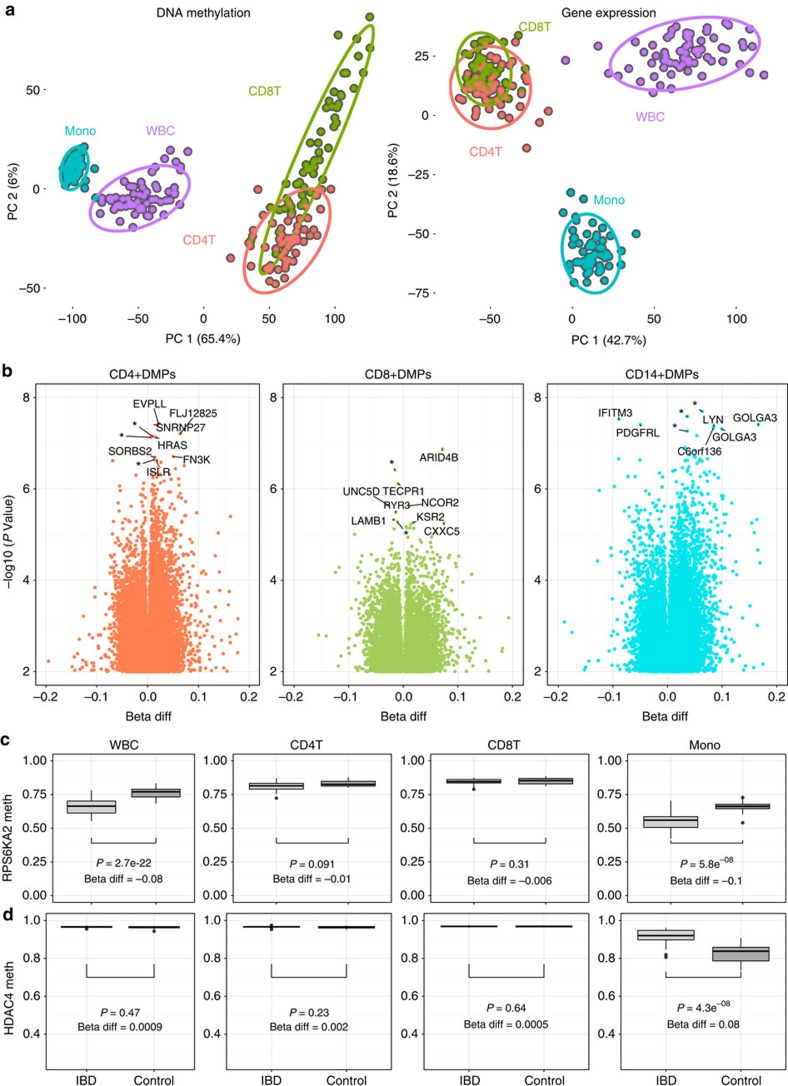
Cell-type specificity of DNA methylation signals. (**a**) Principal component analysis demonstrating the first two components of the entire (i) DNA methylation data set (ii) Gene expression data set. Both demonstrate tight clustering according to the cell type of origin of the sample. (**b**) Volcano plots for IBD versus controls differential methylation position (DMP) analysis for separated cells (CD4^+^, CD8^+^ T cells and CD14^+^ monocytes). (**c**,**d**) Boxplots show the median, 25th and 75th percentiles, and 1.5 * interquartile range (error bars) of methylation (beta) values. Cell-specific DNA methylation profiles. (**c**) The top differentially methylated position (RPS6KA2) was hypomethylated in whole blood and also monocytes. There was a larger difference between cases and controls in the separated cells compared with whole tissue (blood). (**d**) demonstrates monocyte specific DNA methylation at the histone deacetylase 4 (HDAC4) locus. Beta differences and uncorrected *P* values derived from linear models (IBD cases versus controls, including age and sex as covariates).

The cell-specific nature of DNA methylation signatures is well-known. Circulating leukocytes were selected for DNA methylation study as the most disease-relevant tissue in IBD. IBD is an immune-mediated disease with well-recognised extra-intestinal manifestations. Much of the current armamentarium of IBD therapy targets the peripheral immune system. Principal component analysis of the entire cohort of whole blood and separated cell DNA methylation data demonstrates tight clustering according to cell type of the sample (Fig. 3a). The purity of isolated cell populations was assessed using flow cytometry (CD14^+^ median=92.4% (IQR 87–94.9), CD4^+^=97.3% (93.8–98.9), CD8^+^=88.7 (80.5–93)) and *in silico* using a previously described algorithm^17^ used to estimate cell proportions based entirely on methylation data (CD14^+^ median=98.8% (IQR 93.7–100), CD4^+^=98.8 (93.7–100), CD8^+^=87.2 (75.9–91.5)).

Comparisons between IBD cases and controls in specific cell types are summarized in Fig. 3b. Following Holm correction for multiple testing there were 6 DMPs for CD4^+^ cells, 11 DMPs for CD14^+^ cells and no DMPs in CD8^+^ cells. When using a less stringent threshold there were 763 DMPs for CD4^+^ cells, 899 DMPs for CD14^+^ cells and no DMPs in CD8^+^ cells. All comparisons in the individual cell types are available in Supplementary Tables 14–22.

Data derived from separated cells provides insight into cell type of origin of methylation signals identified in whole blood. *RPS6KA2*, the top DMP in whole blood is also hypomethylated in CD14^+^ monocytes (Δβ −11.7%, *P*=5.8 × 10^−8^, false discovery rate (FDR)-adjusted *P*=0.009, linear model, Fig. 3c). The difference in beta values between cases and controls was larger in monocytes compared with whole blood, suggesting that monocytes are likely to be responsible for the signal seen in whole blood. The smaller effect sizes (Δβ) seen in whole blood may represent a ‘dilution' effect given that monocytes make up a small fraction of all leukocytes. Cell-specific data also demonstrates a DMR present in CD14^+^ monocytes in *HDAC4* (3 hypermethylated probes, 1,253 bases, minimum *P* value=4.1 × 10^−7^, minimum FDR-adjusted *P*=0.006, linear model, Fig. 3d). This is particularly interesting given *HDAC4* is a subclass of histone deacetylase enzymes, and may indicate interaction between epigenetic mechanisms.

### Differential methylation may be driven by genetic variants

The IBD-associated DMPs appear to co-localize with known IBD-associated GWAS loci. When compared with randomly generated bins with similar probe density, there was a significant enrichment of DMPs within defined distances of GWAS loci (Bin size 25 kb *P*=0.0012, 50 kb *P*=2.27 × 10^−6^, 100 kb *P*=4.85 × 10^−11^, 250 kb *P*=1.7 × 10^−20^, Supplementary Fig. 10A). This effect appeared to be IBD-specific with no enrichment for other related and non-related complex diseases GWAS loci (Supplementary Fig. 10B).

**Figure 4:**
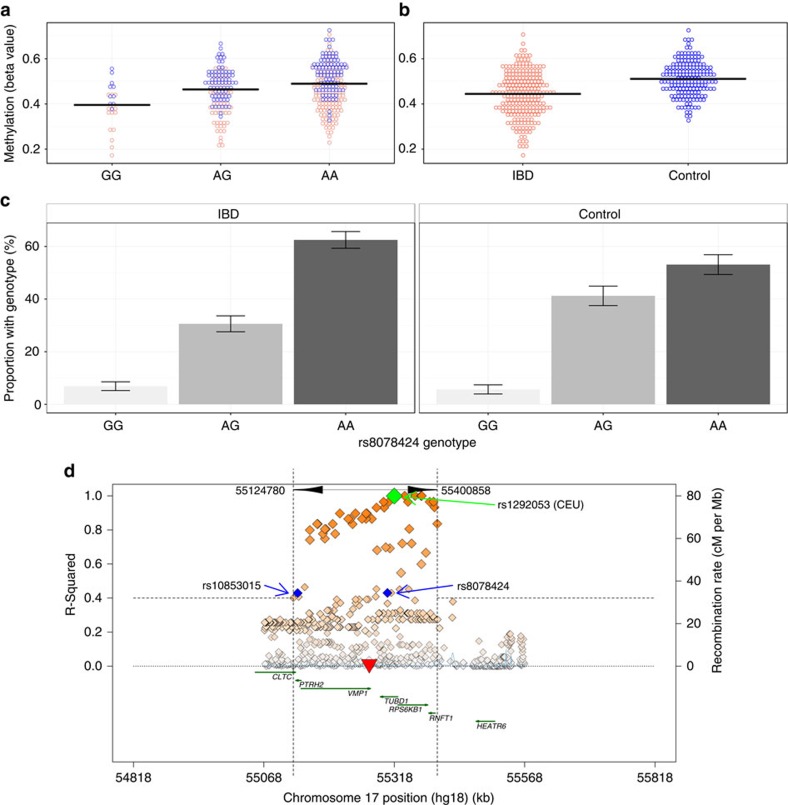
VMP1 genotype associates with DNA methylation. (**a**) The genotype of rs8078424 strongly associates with VMP1 DNA methylation (cg16936953) (FDR corrected *P*=8.8 × 10^−5^, linear model). (**b**) VMP1 DNA methylation strongly associates with Inflammatory bowel disease (IBD) case status compared with controls (Holm corrected *P*=2.2 × 10^−13^, linear model). (**c**) The rs8078424 genotype associates with IBD status (Cochran-Armitage test 1df *χ*^2^=4.7 uncorrected *P*=0.03)(rs10853015 also associates Cochran-Armitage test 1df χ^2^=6.6 uncorrected *P*=0.01, both in Hardy-Weinberg equilibrium). Error bars denote s.e. (**d**) The two SNPs (rs10853015 and rs8078424, blue diamonds) that associate with methylation of the VMP1/microRNA-21 locus (red inverted triangle) are in linkage disequilibrium with the known IBD-susceptibility polymorphism (rs1292053, green diamond)(rs1292053-rs8078424, distance=13072, bp, *D*′=1, *r*^2^=0.43 and rs1292053–rs10853015, distance=185198, *D*′=0.93, *r*^2^=0.43). Plot generated using SNAP^69^.

Given that many of the hitherto described genetic variants do not exist in sequence altering positions, DNA methylation may be an important intermediary between genetics and disease. A previous study attempted to determine DNA methylation as a mediator of genetic risk in rheumatoid arthritis; a similar methodology to which has been applied to the present IBD data set^4^. The 439 IBD-associated DMPs were investigated for local genetic association (*cis* methylation quantitative trait loci (meQTL)). Using a threshold distance of 1 mb, minor allele frequency >0.1 and age, sex and cell proportions as covariates, 326 meQTLs (74 independent DMPs, 292 independent single-nucleotide polymorphisms (SNPs)) were identified. Two of five aforementioned DMRs (*VMP1* and *ITGB2*) have significant genetic associations. Seven DNA methylation probes within the *VMP1*/microRNA-21 locus associate with two SNPs (rs10853015, rs8078424, both in Hardy–Weinberg equilibrium, Fig. 4b,c). These two SNPs are in linkage disequilibrium with a known IBD-susceptibility allele (rs1292053-rs8078424, distance=1,3072, bp, *D*′=1, *r*^2^=0.43 and rs1292053–s10853015, distance=185,198 bp, *D*′=0.93, *r*^2^=0.43, Fig. 4d)^1^,^22^, a finding that offers the tantalizing possibility that the known IBD susceptibility SNP mediates its effect on disease via DNA methylation. While three of the four criteria of causal inference^23^ have been satisfied; we have not been able to demonstrate that *VMP1* methylation mediates between genotype and disease status (genotype is not independent of phenotype following adjustment for methylation)^4^,^5^,^24^. This may reflect insufficient power to identify genetic associations in IBD cases. Nonetheless this demonstrates that IBD-specific differences in methylation may be driven by underlying genetic variants, and provide a potential mechanism by which genetic polymorphisms may contribute to disease. The second DMR with a significant genetic association is integrin subunit beta-2 (*ITGB2*). Three SNPs associated with *ITGB2* methylation are close, but not in linkage disequilibrium with another previously described IBD susceptibility allele (rs7282490).

### Relating cell-specific DNA methylation and gene expression data

**Figure 5:**
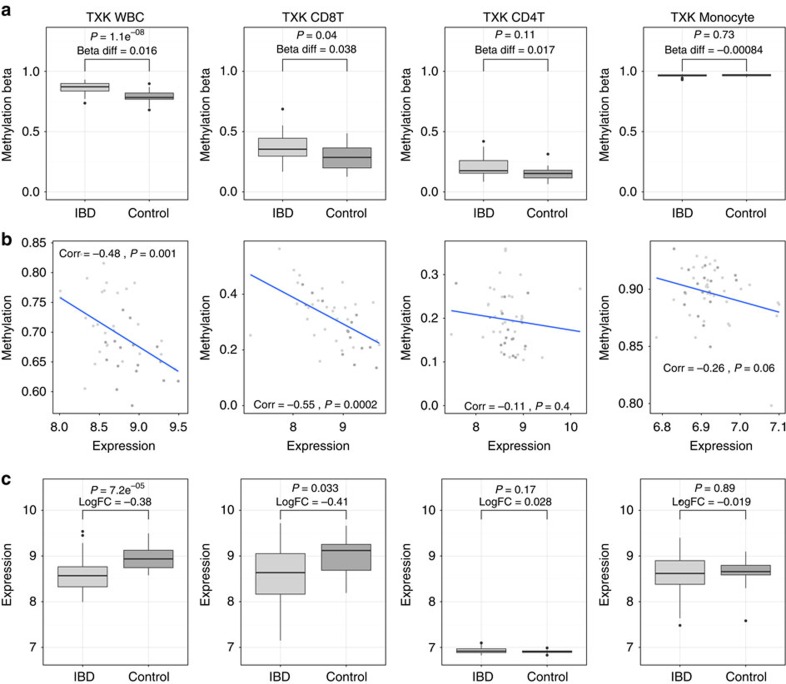
DNA methylation relates with cell-specific TXK gene expression. TXK (Tyrosine Kinase) DNA methylation (**a**) and gene expression (**c**) in whole blood (Globin mRNA depleted), CD8^+^ T Cells, CD4^+^ T Cells and, CD14^+^ monocytes. Differences in methylation (Diff Beta), and gene expression (LogFC, Log fold change) and uncorrected *P* values (*p*) derived from linear models including age and sex as covariates. (**b**) demonstrates correlation (*P* Values derive from Pearson's correlation) between TXK gene expression and DNA methylation in matched samples. Boxplots show the median, 25th and 75th percentiles, and 1.5 * interquartile range (error bars).

The relationship between DNA methylation and gene expression is complex and is likely to be cell specific. CpG island methylation occurring within promoter regions and transcription start sites (TSS) is known to be associated with reduced gene expression^25^. Detailed characterization of the transcriptome in whole blood and separated leukocytes using Illumina HT12 microarrays provided multilevel matched genetic, methylation and expression data for a subset of patients (Supplementary Table 23). IBD-associated hypermethylation within the *TXK* gene between the 5′ untranslated region and first exon region was associated with a reduction in TXK gene expression seen in globin mRNA depleted whole blood (log fold change=−0.38, *P*=7.2 × 10^−5^, linear model) and CD8^+^ T cells (log fold change −0.41, *P*=0.03, linear model), but not other cell types (Fig. 5). Like the DNA methylation data, the difference in gene expression was larger in separated cells compared with whole tissue, indicating the difference seen in the cell type of origin may become diluted within the whole blood signal. There was statistically significant negative correlation between TXK gene expression (ILMN_1741143) and all three DNA methylation probes included in the DMR in whole blood (cg02600394 Pearson's correlation=−0.48 *P*=0.001, cg20981615 corr=−0.49 *P*=0.0007, cg17984638 corr=−0.44 *P*=0.003) and CD8^+^ cells (cg02600394 Pearson's correlation=−0.55 *P*=0.0002, cg20981615 corr=−0.56 *P*=0.0001, cg17984638 corr=−0.7 *P*=2 × 10^−7^) but not for other cell types in matched samples. The level of expression of TXK appears to be similar in T-cells (CD4^+^ and CD8^+^) but lower in monocytes. Using a method that explores methylation within TSS and/or known regulatory regions and gene expression within gene networks has highlighted several functional epigenetic modules of biological relevance that were significantly associated with IBD (Supplementary Table 24)^26^.

### DNA methylation biomarkers offers translational potential

The paired methylation probe biomarkers described in Adams *et al*.^16^ in paediatric CD and controls were prospectively validated in the present adult data set using linear discriminant analysis. The *RPS6KA2*/*VMP1* probes (cg17501210/cg12054453) and *RPS6KA2*/*TNFSF10* probes (cg17501210/ cg01059398) were able to accurately discriminate between disease and control in CD (area under receiver operating characteristic curve (AUC)=0.84/0.81 respectively); IBD (AUC=0.79/0.79) and UC (AUC=0.73/0.71; Supplementary Fig. 11A–C). Novel diagnostic DNA methylation biomarkers were identified in the present cohort by L1 penalised logistic regression (lasso)^27^. The cohort was randomly split into a learning set (2/3 of the cohort=287 individuals) and a testing set (*n*=144). The best-performing model that included 30 methylation probes was able to discriminate between IBD cases and controls with a high degree of accuracy (AUC=0.898, sensitivity=0.812, specificity=0.847, misclassification rate=0.174, shrinkage intensity was a normalization fraction of 0.06, Supplementary Fig. 12). The number of methylation probes included in the model could be reduced to 3 probes (*RPS6KA2* cg175012010, cg09349128, cg25114611); however, this led to a reduction in specificity for IBD and a higher misclassification rate (AUC=0.87, sensitivity=0.906, specificity=0.542, misclassification rate=0.243). Similar discriminatory ability could be obtained for CD versus control (AUC=0.89, 42 probes) and UC versus control (AUC=0.81, 12 probes). Clinically it can be difficult to distinguish CD from UC; a 19-probe panel was able to distinguish CD from UC with a good degree of accuracy (AUC=0.719, sensitivity=1) using the same method.

The most pressing clinical need is the development of biomarkers capable of predicting disease course. Lee *et al*.^28^ used CD8^+^ T-cell transciptomic data to identify subclasses of IBD patients with more and less severe disease courses. We applied a similar unsupervised consensus clustering methodology^29^ to the top 5,000 most differentially methylated 450K probes (IBD versus control analysis, whole blood) in the IBD cases only. Methylation data clustered into three stable clusters (Supplementary Fig. 13, determined using clest^30^ and cumulative distribution functions^31^). Univariate survival analysis demonstrated that the three subclasses were associated with high-, moderate- and low-risk of requiring surgery (resection or colectomy, *χ*^2^-test for difference between survival curves with 2 degrees of freedom *P*=0.01), emergency hospital admission (*P*=0.0008) and immunomodulatory therapy (oral or IV steroids, azathioprine, anti-tumour necrosis alpha monoclonal therapy, ciclosporin, methotrexate, *P*=0.02, Supplementary Fig. 14A–D). Cox proportional hazards regression including other clinical covariates (age, sex, CRP, albumin and haemoglobin) demonstrated that the IBD subclasses were not independently predictive of outcome (Supplementary Table 25).

## Discussion

This study has demonstrated site-specific methylation changes in IBD compared with controls that were strongly significant following stringent correction for multiple testing. In lieu of a consensus on an accepted significance threshold for EWAS, a correction method has been used here as has traditionally been applied to GWA data. Using this conservative threshold, 439 significant DMPs and 5 DMRs have been identified. Whereas many early EWAS results have not been replicated, the highly replicable nature of DMPs and DMRs in independent cohorts in this study increases the confidence in the findings. A comprehensive approach was employed to study genome-wide DNA methylation, using whole genome bisulphite sequencing, Illumina 450K arrays and pyrosequencing allied with genomic and transcriptomic data in matched individuals allowing truly integrative analysis.

The top IBD-associated DMR, *VMP1* (vaculole-membrane protein 1), was also one of the most significant DMPs. *VMP1* was also the principal finding in our previous paediatric study, and is validated here in a significantly larger cohort. The majority of methylation probes in the *VMP1* area are found towards the 3′-end of the *VMP1*, which coincides with the primary transcription site for microRNA-21 (pre-miR21). This is a promising avenue for further research given the pro-inflammatory status of microRNA-21 and its previous implication in colitis and IBD pathogenesis^32^,^33^. Another notable IBD-associated DMR is *ITGB2* (integrin subunit beta 2), the gene of which has a role in leukocyte adhesion, activation and trafficking^34^. This is particularly interesting given the recent focus on strategies to therapeutically target leukocyte adhesion, namely vedolizumab, which targets a different integrin subunit (α4β7)^35^. Aberrant DNA hypermethylation at the *ITGB2* locus has previous been demonstrated in IBD in mucosal^14^ and peripheral blood leucocyte^15^ samples as well as in other diseases^36^. The other DMRs are also of great interest: *WDR8* or *WRAP73* (WD (trp-asp) repeat protein family, antisense to Trp73) which is involved in several cellular and gene regulatory processes^37^ and *TXK* is discussed in below.

Whilst DMRs have been considered as the hallmarks of differential methylation, DMPs should also not be overlooked. The top DMP was *RPS6KA2*, a ribosomal kinase in the serine/threonine kinase family^38^ that regulates a diverse set of cellular processes including cell growth, cell motility, proliferation and cell cycle progression^39^. *RPS6KA* is involved in several stages of translational control and is a mediator in the PI3K/Akt/mTor pathway^39^. mTOR is involved in autophagy, which importantly is dysregulated in CD. Given that IBD-associated aberrant *RPS6KA2* methylation occurs within the gene body at a region with dense CpG content, and that no difference was seen in RPS6KA2 gene expression, the functional relevance of this finding may be difficult to delineate. *RPS6KA2* is found within an IBD-associated GWAS locus (rs1819333, *P*=6.76 × 10^−21^, odds ratio=1.08)^22^, and it is possible that a genetic polymorphism may be contributing to this strong finding. *RPS6KA2* methylation has previously been associated with cigarette smoking^20^, while it appears that smoking status does not account for the disease-associated methylation difference in this data set, is interesting given that smoking is a known environmental modifier of IBD. The protein encoded by *SBNO2*, Strawberry notch homologue 2, another DMP, is known to have an anti-inflammatory effect by acting as part of the interleukin-10 downstream pathway^40^. Again, *SBNO2* is found within an IBD-associated GWAS locus^41^. Other highly interesting DMPs implicated in well-known IBD pathogenic pathways include interleukin-23 subunit A (*IL23A*), another IBD GWAS-susceptibility locus, and tumour necrosis factor superfamily member 10 (*TNFSF10*/*TRAIL*).

The impact of cellular heterogeneity on DNA methylation data is a commonly cited limitation of EWAS studies conducted using whole tissues^42^. Statistical algorithms that can provide estimated cell proportions and allow adjustment for cellular heterogeneity are now widely performed throughout the EWAS literature^17^. There are comparatively fewer epigenetic studies with separated cell data, particularly disease-relevant cells and this is a significant strength of the present study^43^. Detailed characterization of matched genetic, DNA methylation and expression data in separated leukocytes has highlighted several cell-specific findings. For example, the top DMP in whole blood, *RPS6KA2*, was differentially methylated in CD14^+^ monocytes but not CD4^+^ or CD8^+^ lymphocytes, potentially providing insight into the cell type of origin of methylation signals seen in whole tissue. These results must be interpreted with some caution due to the different proportions of cells in IBD compared with non-IBD. Cell specificity may become even more relevant when analysing the relationship between methylation and gene expression. A major finding of this study is IBD-associated hypermethylation of the *TXK* TSS occurring specifically within CD8^+^ cells, with an appropriate negative correlation with decreased gene expression in CD8^+^ T cells of the same individual. Expression of TXK, a member of the Tec family of non-receptor tyrosine kinases, in Th1 T cells is obligatory for the production of interferon gamma^44^. CD8^+^ T cells have an established role in IBD pathogenesis^28^,^45^,^46^ with recent data suggesting that CD8^+^ T cell exhaustion may be a critical prognostic factor in immune-mediated diseases^47^.

While this observational study design does not allow functional interrogation of the origin of the DNA methylation profile seen here in IBD, it is interesting to speculate on the origin of such signals. The strong correlation between clinical inflammatory markers and the top DMPs and DMRs perhaps indicate that the observed methylation changes are a consequence of inflammation. It is notable that these signals endure in the replication cohort that consists of patients with established disease sampled following treatment. A small study of mucosal DNA methylation in children with UC suggests that the methylome reverted back towards that of healthy controls following treatment^14^. Contrary to this hypothesis is the strong association between germ-line variation and methylation of two of the five DMRs (*VMP1*/microRNA-21 and *ITGB2*). This exciting novel finding associates methylation in the *VMP1*/microRNA-21 region with two nearby SNPs, which act as methylation quantitative trait loci (meQTLs). These meQTLs (rs10853015, rs8078424) are in linkage disequilibrium with a known IBD-susceptibility GWAS locus (rs1292053). DNA methylation may be a mechanism by which genetic variants outside of protein coding regions may contribute to the disease phenotype. In rheumatoid arthritis, Liu *et al*.^4^ used mediation analyses to demonstrate that methylation was the causal mechanism by which genotype conferred disease risk. Most associations occurred in the major histocompatibility complex region, known to harbour many genetic variants with complex and extended linkage disequilibrium structures^48^. In the present study we were able to establish several of the principals of causal inference but not independence of genotype and phenotype following adjustment for methylation, a finding reflected in the complex disease literature^5^. The strong association between top ranking IBD-associated DMP/DMR and nearby genetic variants nevertheless represents a major finding and goes some way to explain the significant site-specific methylation differences in IBD cases and controls.

DNA methylation data offer immediate translational potential as biomarkers. The *SEPT09* blood-based DNA methylation biomarker has been used in diagnosis and screening for colorectal cancer^49^. We have previously demonstrated two methylation probes can accurately differentiate CD and controls and have prospectively validated the same two probe markers using the present data set^16^. Here we have used lasso^27^, an established machine learning technique that can help avoid over-fitting in large data sets where the number of variables vastly exceed the number of samples. The final 30-probe model is easily scalable into a high-throughput pyrosequencing panel. Such a non-invasive peripheral blood biomarker could be used to stratify patients to further intrusive investigations (e.g., colonoscopy). Existing clinically available biomarkers such as faecal calprotectin^50^ already provide similar utility but are unable to distinguish the two forms of IBD. A different 19-probe methylation-based panel can discriminate CD and UC, potentially facilitating clinical decision-making where the medical and surgical management of the two diseases differ. Currently no prognostic biomarkers can reliably differentiate patients requiring early aggressive treatment from those who would experience a quiescent disease course. There has been some anticipation that emerging ‘-omic' data may provide such a biomarker^28^. We have identified a DNA methylation signature that associates with high-, intermediate- and low-risk of specific deleterious outcomes. Consensus clustering has recognized limitations^51^ and it is noteworthy that the methylation subclasses are not independently predictive of outcome and are likely to be driven by underlying differences in cell count or other clinical parameters.

The primary whole-blood analysis was well powered to detect differential methylation between IBD cases and controls and key findings were replicated in a similarly sized replication cohort using an orthogonal technique. The allied separated cell DNA methylation, gene expression and genetic data sets provided complimentary data and allowed further investigation of key findings from the primary analyses but were not sufficiently powered to provide meaningful conclusions in their own right. The cross sectional study design limits the ability to define cause and effect, in particular, the contribution of genotype and inflammation. The functional implications of methylation changes identified here may be investigated using recently developed CRIPSR technology^52^. Samples from patients with different complex inflammatory diseases would help define the specificity of described methylation changes to IBD. While the impact of cellular heterogeneity on whole-tissue methylation is well known, existing^17^ and new^53^ algorithms used to estimate cell count are now widely used to adjust for differential cell distribution. Whilst generally accepted, cell fraction predictions are estimates and when included as independent covariables in linear modelling may result in inflated *P* values and potentially false-positive DMPs. We have taken the additional step of performing cell sorting of target tissue to provide insights into cell type of origin of whole-tissue signals. In this study, three cell types have been isolated but it would also be informative to generate data on specific T-cell subpopulations (for example, T-reg cells, naive CD4^+^ cells), other common blood cell types (for example , neutrophils) as well as gut mucosa.

This is the most detailed characterization of the circulating IBD methylome to date. Highly statistically significant and replicable DNA methylation differences have been demonstrated at sites pertinent to disease pathogenesis. DNA methylation may be a factor of underlying germ line variation and may represent a mechanism by which genetic polymorphisms contribute to disease variance. Cell sorting of disease-relevant immune cells has highlighted subtle cell-specific relationships between DNA methylation and gene expression. The immediate agenda for epigenetic research in IBD includes further well powered EWAS in diverse populations, delineating the methylome at the mucosal level particularly in specific gut cell types, and prospective diagnostic and prognostic biomarker studies to facilitate early clinical translation.

## Methods

### Patient selection and ethics

To limit the potential effect of chronic inflammation and powerful immunomodulatory drugs on the epigenetic profile, patients were recruited as close to diagnosis as possible. The patients were recruited prospectively as part of the IBD–BIOM inception cohort from gastroenterology and endoscopy appointments.

Symptomatic controls were recruited from gastroenterology clinics during the same period; following rigorous investigation these individuals were found not to have IBD or any other organic bowel pathology. A further control group consisting of healthy volunteers with no self-reported gastrointestinal symptoms were also recruited.

The Tayside Committee on Medical Ethics B granted approval for this study with all patients and controls giving written, informed consent (LREC 06/S1101/16, LREC 2000/4/192).

### Sample collection and immunomagnetic cell sorting

Blood samples were taken in the following tubes: 9 ml Z Serum clot activated vacuette (Greiner, Frickenhausen, Germany), 9 ml K3 EDTA vacuette (Greiner) and PAXgene blood RNA tubes (BD, NJ, USA). Between of 18–36 mls of EDTA buffered blood was used for an initial Ficoll (Ficoll-Paque, GE healthcare, Bucks, UK) density gradient centrifugation to obtain peripheral blood mononuclear cells. Cells labelled with antibody-coated microbeads (human CD14^+^, CD8^+^ and CD4^+^ microbeads, 20 μl per 1 × 10^7^ cells) were immunomagnetic separated using the autoMACs Pro cell separator (Miltenyi, Germany). CD4^+^ separations were performed following an initial CD14^+^ depletion step. Cell purity was estimated using florescent antibody staining and flow cytometry (FACS Aria II, BD, Germany). Following peripheral blood mononuclear cell depletion, erythrocytes were lysed on ice (1,000 ml dH_2_O, 8.3 g of NH_4_Cl, 1.0 g of KHO_3_ and 1.8 ml of 5% EDTA) and granulocytes were recovered by centrifugation.

### Genome-wide methylation profiling

Peripheral blood leukocyte DNA was bisulphite converted and analysed using the Illumina HumanMethylation450 platform (Illumina, San Diego, CA, USA)^54^. Cases, controls and different cell types were randomly distributed across chips. Data were processed using the lumi^55^, methylumi^56^ and minfi^57^ packages in R (R Foundation for Statistical Computing, Vienna). Probes were filtered out if the detection *P* value of ≥0.01 or if >5% of probes failed. Probes containing SNPs with a minor allele frequency of ≥0.01 in the European population in the 1000 Genomes Project were also removed^58^. Samples with >5% of probes failing and those failing a sex check (based on X chromosome methylation level) were also removed. In methylumi, probes were background adjusted, corrected from dye colour bias, and quantile normalized. Intra-array and probe design variation was corrected for using beta-mixture quantile dilatation (BMIQ)^59^. Inter-array batch effects were corrected for using ComBat^60^. Cell proportions were estimated from methylation data using the Houseman algorithm^17^ as implemented in minfi. Differentially methylated position analysis (DMP, single CpG probe) was performed using limma using the aforementioned cell proportions together with age and sex as covariates^61^. Statistical significance was set at *P*<0.05 following adjustment for multiple testing using Holm correction^18^ for whole-blood data, and the Benjamini Hochberg FDR^62^ for separated cell data.

Differentially methylated regions (DMRs) were defined as three or more contiguous probes within a 2 kb distance, each sharing the same direction of methylation change, each achieving a Holm corrected *P*<0.05 in DMP analysis. A previously described^16^ reimplementation of the Lasso function from the CHaMP pipeline^63^ was used for this analysis (Please note that the Lasso function described here is distinct from the lasso function described below in the biomarker discovery section).

A power calculation was performed based on the data from our previous paediatric study^16^. The median transformed standard deviation was calculated across the most variable 1000 probes (median standard deviation=0.02, Supplementary Fig. 15A,B). Curves (Supplementary Fig. 15C) were plotted using the pwr.t.test function for varying n, with an alpha value of genome wide significance was set at *P*=1 × 10^−7^ with the beta error along the y axis (ab line at 80%) and effect size (d) along the *x* axis. For an 80% power to detect an effect size of a difference in means of one s.d. was 100 patients per group.

The proximity of differentially methylated probes to the 163 known IBD-associated GWAS risk loci described in Jostins *et al*.^22^ within range thresholds of 25, 50, 100 and 250 kb was compared with 1,000 randomly selected bins of the same size with matched probed density using Wilcoxon rank sum test. The same methodology was utilized in our previous study^16^. As a control, the IBD-associated DMPs were also tested for enrichment with GWAS data obtained from the GWAS catalogue (http://www.genome.gov/gwastudies/) for seven other diseases; rheumatoid arthritis, psoriasis, ankylosing spondylitis, TB, type I diabetes, Alzheimer's disease, IgG glycosylation, colorectal cancer and hair colour.

### Whole genome bisulphite sequencing

Whole genome bisulphite sequencing was performed on six IBD cases (3 CD, 3 UC) and three controls using a similar method to that described elsewhere^64^. Whole blood genomic DNA (1–2 μg) was spiked with λ DNA (5 ng of λ DNA/microgram of genomic DNA; Promega). DNA was sonicated to create fragments of 50–500 bp in size, and fragments of 150–300 bp were size-selected using AMPure XP beads (Agencourt Bioscience). DNA libraries were created using the Illumina TruSeq Sample Preparation kit following Illuminas standard protocol: DNA fragments underwent end repair, an adenine was added to the 3′ end and Illumina TruSeq adaptors were ligated to each end. Following adaptor ligation, DNA was twice bisulfite converted using the EpiTexy Bisulfite kit (Qiagen), following the manufacturer's protocol to obtain a conversion rate >99%. PCR using PfuTurboCx Hot-Start DNA polymerase (7 cycles, Stratagene) was performed to enrich samples for adaptor-ligated DNA. The library was quality assessed using the Agilent 2100 Bioanalyzer, and the concentration estimated using the library quantification PCR kit (Kapa Biosystems). The Illumina HiSeq 2000 platform was used to perform paired-end DNA sequencing (2 × 100 bp).

A previously described pipeline for data processing to a final data set of called CpG files was performed at Centro Nacional de Análisis Genómico^64^. Briefly, reads were mapped using the GEM aligner (v1.242) against two versions of the human GRCh37 reference genome (hg19, version 1—C replaced by T, version 2 G replaced by A) with the original sequence being stored. Up to four mismatches per read with respect to the reference were allowed. After read mapping, the original sequence for each read was restored.

Estimation of cytosine levels was carried out on read pairs where both members of the pair mapped to the same contig with consistent orientation and there was no other such configuration at the same or a smaller edit distance from the reference. After mapping, we restored the original read data in preparation for the inference of genotype and methylation status. We estimated genotype and DNA methylation status simultaneously taking into account the observed bases, base quality scores and the strand origin of each read pair. For each genome position, we produced estimates of the most likely genotype and the methylation proportion (for genotypes containing a cytosine base on either strand). A Phred-scaled likelihood ratio for the confidence in the genotype call was estimated for the called genotype at each position. For each sample, CpG sites were selected where both bases were called as homozygous CC followed by GG with a Phred score of at least 20, corresponding to an estimated genotype error level of ≤1%. Suspected centromeric or telomeric repetitive regions characterized by >500 × coverage depth were excluded. A common set of called CpG sites was generated, and used in subsequent analyses.

### Pyrosequencing

There were 28 overlapping cases used in our previous study (Adams *et al*.) that were used in addition to 310 independent samples. DNA was bisulphite converted using the EZ-96 DNA Methylation kit (Zymo Research, Irving CA) according to manufacturer instructions. Bisulphite converted DNA was amplified for target sequences using PCR (PyroMark PCR kit, Qiagen, Dusseldorf, Germany). Primers designed using the PyroMark Q24 Assay design software (Version 2.0, Qiagen) and supplied by Sigma Aldrich (St Louis, MS, Supplementary Table 26). Pyrosequencing was performed using the PyroMark Q24 platform and initial data analysis was performed using PyroMark Q24 software (Version 2.0.6.20, Qiagen). Samples were run in duplicate and results with a coefficient of variation of ≥10% were discarded.

### Genotyping

Whole blood leukocyte DNA was extracted using the Nucleon BACC 3 DNA extraction kit (GE healthcare, Buckinghamshire, UK). Patients were genotyped using the Illumina Human CoreExome BeadChip microarray (Illumina, San Diego, CA, USA) and genotypes called by GenomeStudio were used. A sex-check was performed using plink to identify and remove sex-mismatches. MeQTLs and eQTLs were estimated using the matrixEQTL packages^65^.

### Gene expression analysis

In addition to the subset of individuals with detailed separate cell samples (*n*=60), a further eight patients were included in gene expression array analyses (total *n*=68). Separated cell RNA was extracted using the Allprep DNA/RNA miRNA universal kit (Qiagen). Whole blood RNA was extracted from PAXgene tubes using the PAXgene blood miRNA kit (PreAnalytix, Switzerland). The RNA was quantified and assessed for quality using the Agilent BioAnalyzer with only samples with a RNA-integrity number of >7 being used for downstream analyses. Following sample concentration and cleanup using the MinElute RNA cleanup kit (Qiagen), globin mRNA transcripts were depleted using GlobinClear (Ambion, Life Technologies USA). RNA was amplified and biotylated using the Illumina TotalPrep RNA Amplification Kit (Ambion, Life Technology). The cRNA was quantified and assessed for quality using the Agilent BioAnalyzer with the expected gel appearance of cRNA is a ‘smear', with a distribution of cRNA size is expected between 250–5,500 nucleotides, with most cRNA between 1,000 and 1,500 nt. Illumina HT12 human v4 expression microarrays were performed using a hybridization time of 18 h at 58 °C. Data were analysed using the lumi^55^ and limma^61^ packages. Data were background adjusted, variance stabilized and quantile normalized.

### Biomarker validation and new biomarker discovery

Biomarkers identified in Adams *et al*.^16^ were validated in this new adult data set using the same methodology (linear discriminant analysis) and the previously published methylation probe pairings. Two new methods were used for biomarker discovery. The CMA package^66^ package aims to address the situation whereby the number of variables vastly outnumbers the number of samples, common in microarray studies. Fully Pre-processed beta values were used to discriminate between IBD cases and controls. Using the CMA package, the available methods of variable selection were assessed. On the basis of AUC, Lasso (least absolute shrinkage and selection operator^27^,^67^) was the best performing variable classification method. The LassoCMA function was used to perform the lasso algorithm for shrinkage and selection of methylation probes to be used as putative biomarkers. The cohort was arbitrarily split into a learning set (2/3 of the cohort=287 individuals) and a testing set of 144 individuals. The L1 shrinkage intensity was tuned to provide the most accurate model, based on the AUC. This involved altering the shrinkage intensity (that is, altering the number of CpG probes that algorithm could include in the model). The random seed was fixed to provide reproducible results. For CD versus UC the learning set was increased to include 3/4 of the cohort.

The second method employed unsupervized consensus (hierarchical) clustering^31^ of median beta values of the top 5,000 DMPs identified in the primary analysis (IBD versus controls, whole blood) using the ConsensusClusterPlus package^29^,^68^. Kmeans clustering of the Pearson correlation coefficient was used as the final clustering method, although similar results were obtained by using other methods (kmeans clustering based on Spearman's correlation, hierarchical clustering, PAM clustering). The number of stable clusters was assessed using the cumulative distribution function (CDF)^31^ and the clest method^30^. Logistic regression was used to compare individuals classified according to clusters and clinical outcomes including need for surgery (intestinal resection and/or colectomy), emergency hospital admission, time until immunomodualtor requirement (IV/oral steroid, thiopurine, ciclosporin, anti-TNFalpha monocolonal antibody therapy, methotrexate) and escalation of therapy as previously defined^28^.

### Data availability

Data have been deposited in GEO as a data series (Accession code GSE87650). All other data are available from the authors on request.

## Additional information

**How to cite this article:** Ventham, N. T. *et al*. Integrative epigenome-wide analysis demonstrates that DNA methylation may mediate genetic risk in inflammatory bowel disease. *Nat. Commun.* **7,** 13507 doi: 10.1038/ncomms13507 (2016).

**Publisher's note**: Springer Nature remains neutral with regard to jurisdictional claims in published maps and institutional affiliations.

## Supplementary Material

Supplementary Information Supplementary Figures 1-15, Supplementary Tables 1-26 and Supplementary References.

## Figures and Tables

**Table 1:** Top table of differentially methylated positions (DMPs) between inflammatory bowel disease (IBD) cases and controls in whole blood.

**Illumina 450K probe ID**	**Chr**	**Gene symbol**	**Feature**	**Relation to UCSC CpG island**	**Δβ**	***P*** **value**	**Holm adj.** ***P*** **value**
cg17501210	chr6	RPS6KA2	Body		−0.08	2.71E−22	1.22E−16
cg18608055	chr19	SBNO2	Body		−0.07	2.02E−20	4.53E−15
cg16936953	chr17	VMP1	Body		−0.09	1.33E−19	1.99E−14
cg09349128	chr22	NA	IGR	N_Shore	−0.04	3.11E−19	3.48E−14
cg25114611	chr6	NA	TSS1500	S_Shore	−0.04	1.10E−18	8.79E−14
cg12170787	chr19	SBNO2	Body		−0.04	1.18E−18	8.79E−14
cg12992827	chr3	NA	IGR		−0.06	6.26E−18	4.01E−13
cg19821297	chr19	NA	IGR	S_Shore	−0.06	3.66E−17	1.98E−12
cg12054453	chr17	VMP1	Body		−0.07	3.98E−17	1.98E−12
cg01059398	chr3	TNFSF10	Body		−0.05	1.59E−16	7.13E−12
cg26470501	chr19	BCL3	Body	S_Shore	−0.03	5.79E−16	2.29E−11
cg07398517	chr3	NA	IGR		−0.04	6.14E−16	2.29E−11
cg26804423	chr7	ICA1	Body		0.04	6.84E−16	2.36E−11
cg18942579	chr17	VMP1	Body		−0.05	1.17E−15	3.74E−11

Adj., adjusted; Chr, chromosome; IGR, intergenic region, TSS1500, within 1,500 bp of a transcription start site.

Δβ, difference in beta values (ratio of methylated and total probe intensity (0 to 1) between IBD cases and controls, positive value indicated increased methylation in cases compared to controls, negative values indicated hypomethylation in IBD cases versus controls. NA denotes methylation probes with no annotated gene symbol. *P* values are derived from linear models including age, sex and cell proportions as covariates.

**Table 2:** List of differentially methylated regions (DMRs) between inflammatory bowel disease (IBD) cases and controls in whole blood.

**Gene**	**Feature**	**Chr**	**Δβ**	**Min Holm adj.** ***P*** **value**	**DMR size**	**Probe counts**	**Disease**
*VMP1*	Body	17	−0.09	5.96E−14	1150	4	IBD, CD, UC
*WDR8*	Body	1	0.03	9.76E−08	1943	3	IBD, CD, UC
*NA*	IGR	1	0.04	1.83E−07	1997	3	IBD
*ITGB2*	5′-UTR	21	0.04	3.28E−05	623	3	IBD, CD
*TXK*	5′-UTR	4	0.02	0.00014	538	3	IBD

Adj., adjusted; CD, Crohn's disease; Chr, chromosome; UC, ulcerative colitis.

Where a single *P* value or beta difference is presented, this represents the corresponding values from the most significant probe within the DMR on differential methylated position analysis (see Table 1). Δβ, difference in beta values (ratio of methylated and total probe intensity (0 to 1) between IBD cases and controls, positive value indicated increased methylation in cases compared with controls, negative values indicated hypomethylation in IBD cases versus controls. NA denotes methylation probes with no annotated gene symbol.
