# Supplementary material for: Integrative epigenome-wide analysis demonstrates that DNA methylation may mediate genetic risk in inflammatory bowel disease
Source: Nat Commun. 2016 Nov 25;7:13507. doi: 10.1038/ncomms13507 (PMC5133631; doi:10.1038/ncomms13507)
Supplement: Supplementary Information — Supplementary Figures 1-15, Supplementary Tables 1-26 and Supplementary References. [file ncomms13507-s1.pdf]

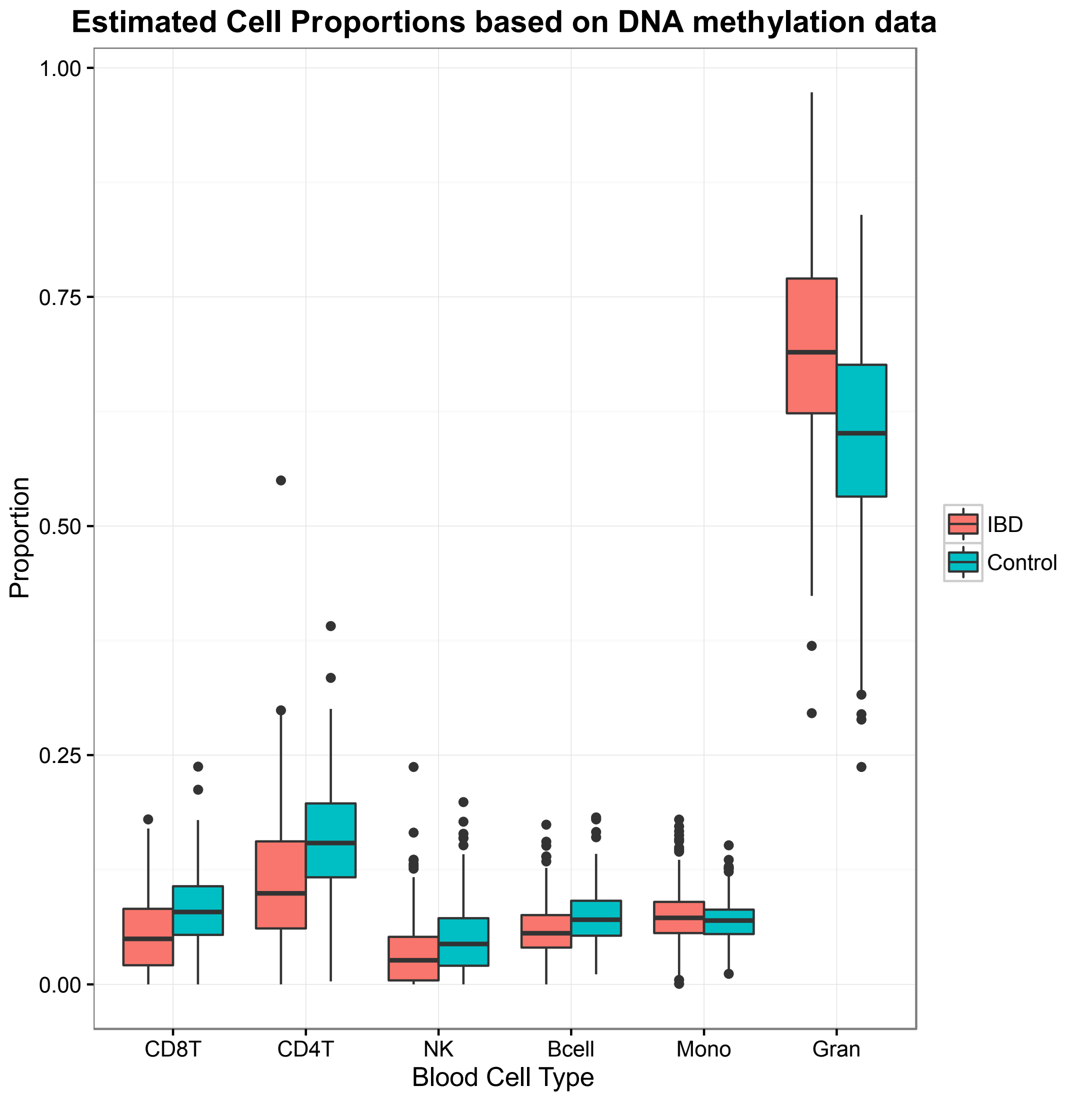


*Supplementary Figure 1 - Estimated cell proportions as derived from Houseman[1] cell mixture deconvolution from DNA methylation data.CD8T=CD8$^+$ T-cells, CD4T=CD4$^+$ T-cells, NK=Natural-Killer Cells, Bcell=B-cell, Mono=Monocyte, Gran=Granulocytes, IBD=inflammatory bowel disease.*

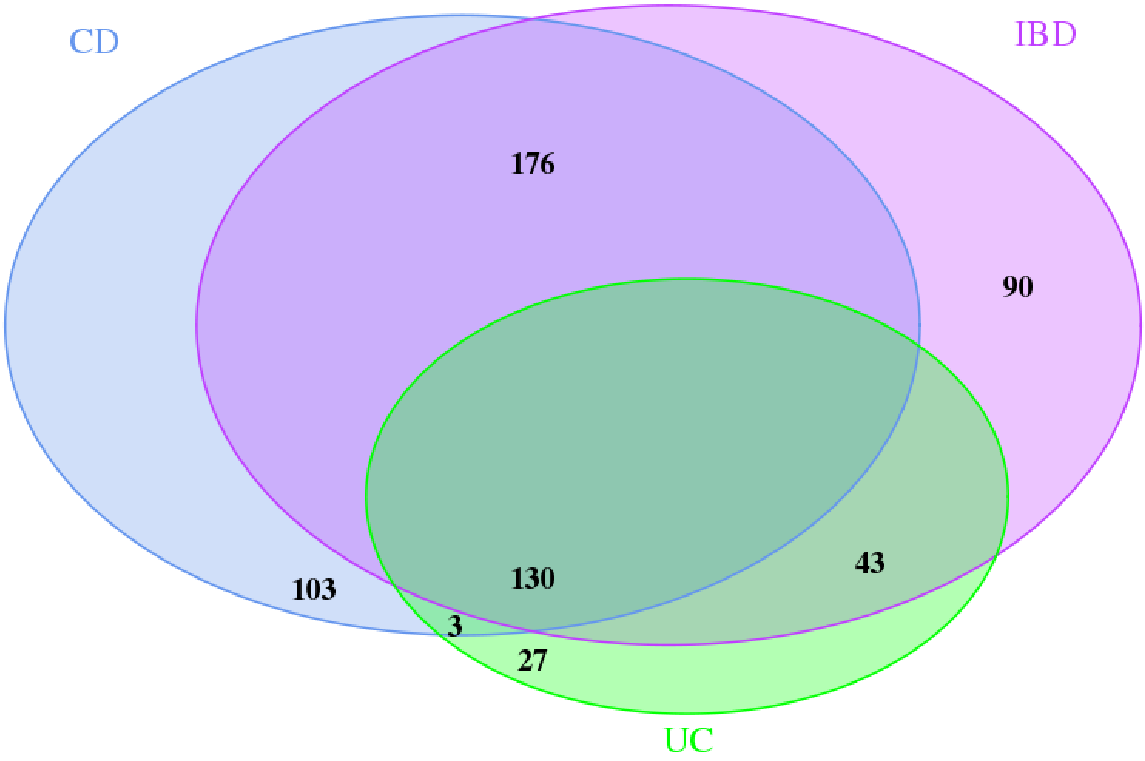


*Supplementary Figure 2 – Venn diagram demonstrating overlap in differentially methylated positions (DMPs) seen in inflammatory bowel disease (IBD, purple), Crohn's disease (CD, blue) and ulcerative colitis (UC, green) versus controls.*

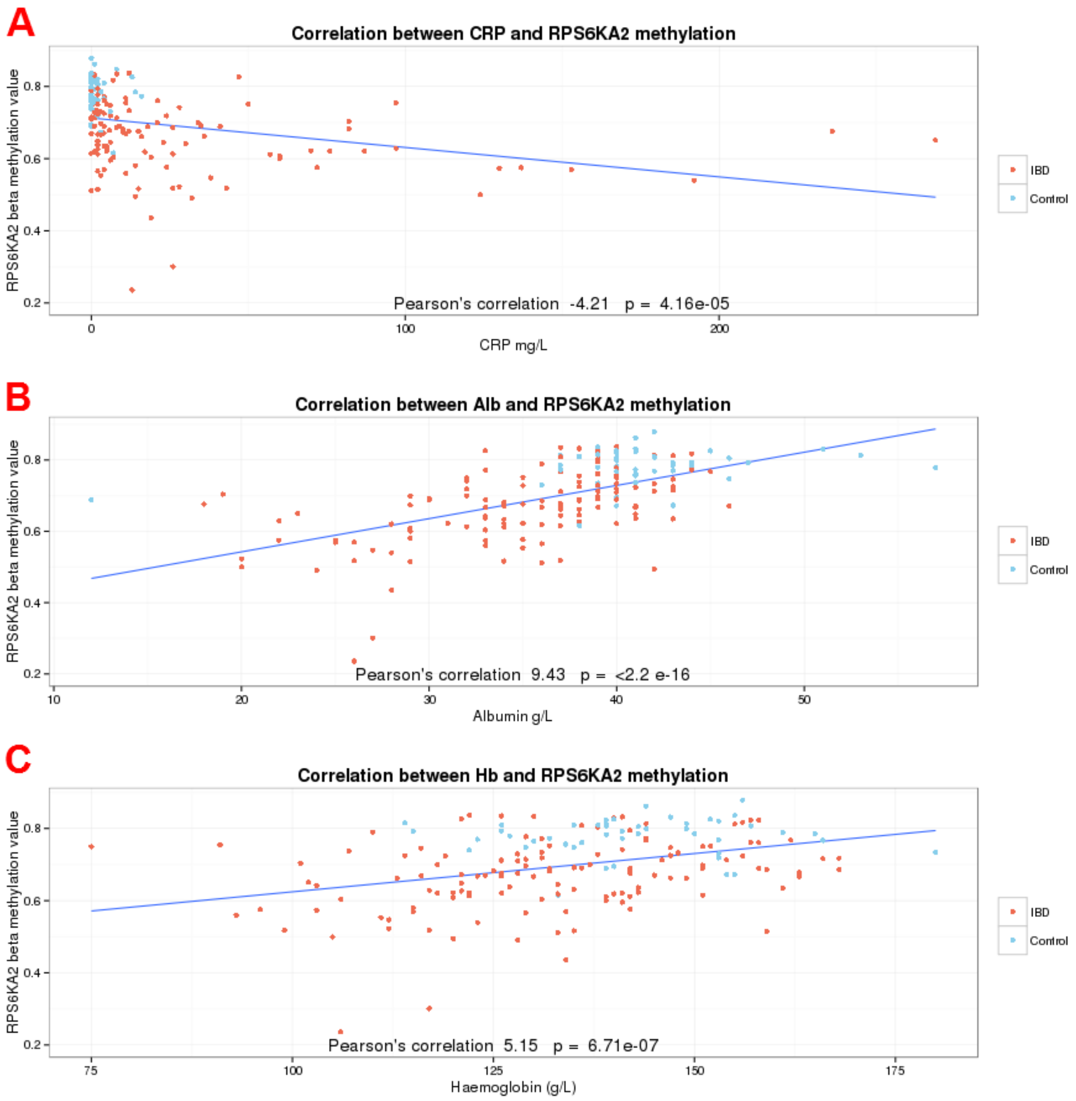
A
Correlation between CRP and RPS6KA2 methylation
RPS6KA2 beta methylation value
Pearson's correlation -4.21 p = 4.16e-05
CRP mg/L
IBD
Control
B
Correlation between Alb and RPS6KA2 methylation
RPS6KA2 beta methylation value
Pearson's correlation 9.43 p = <2.2 e-16
Albumin g/L
IBD
Control
C
Correlation between Hb and RPS6KA2 methylation
RPS6KA2 beta methylation value
Pearson's correlation 5.15 p = 6.71e-07
Haemoglobin (g/L)
IBD
Control

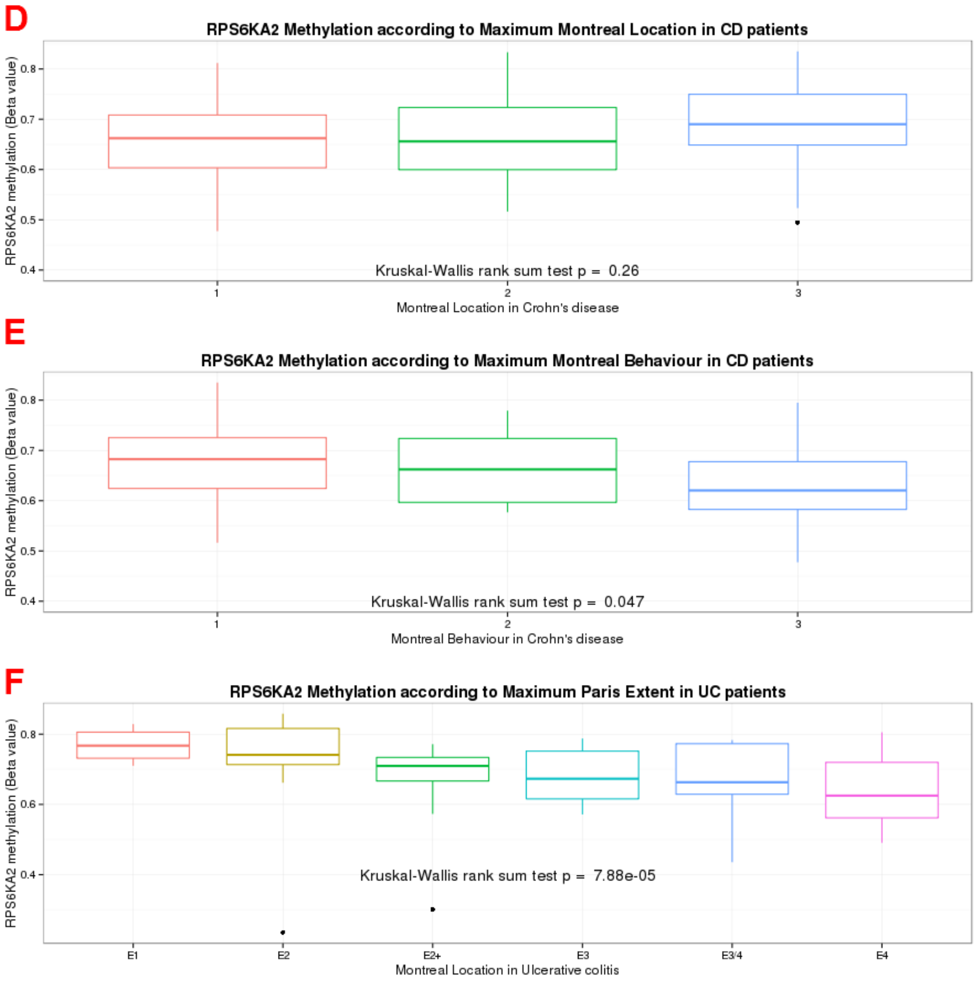


*Supplementary Figure 3 - Correlation between RPS6KA2 methylation (beta value) and CRP (A, mg/L), Albumin (B, g/L) and Haemoglobin (C, Hb, g/L). Correlation and p values denote Pearson's test. Boxplots demonstrating RPS6KA2 methylation with maximum Montreal location in Crohn's disease (D, L1=Ileal disease, L2=Colonic disease, L3=Ileocolonic disease), Montreal behaviour in Crohn's disease (E, B1=inflammation only, B2=structuring disease, B3=penetrating disease) and Montreal Extent in Ulcerative colitis (F, E1=proctitis, E2 = left colonic disease, E3=extensive disease, E3/E4=extends beyond splenic flexure, E4=pancolitis). Statistical test = Kruskall Wallis rank sum test.*

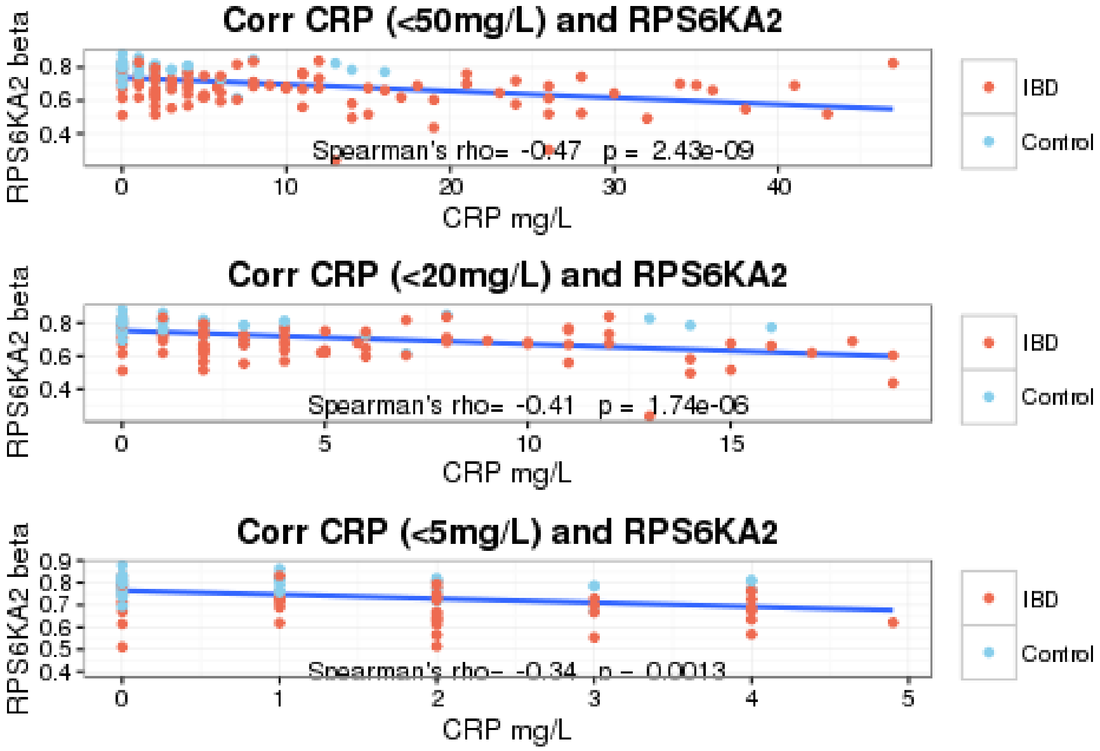


*Supplementary Figure 4 - Correlation between C-Reactive Protein (CRP) and RPS6KA2 beta methylation at different ranges of CRP concentration. Correlation and p value denote Spearman's rank test.*

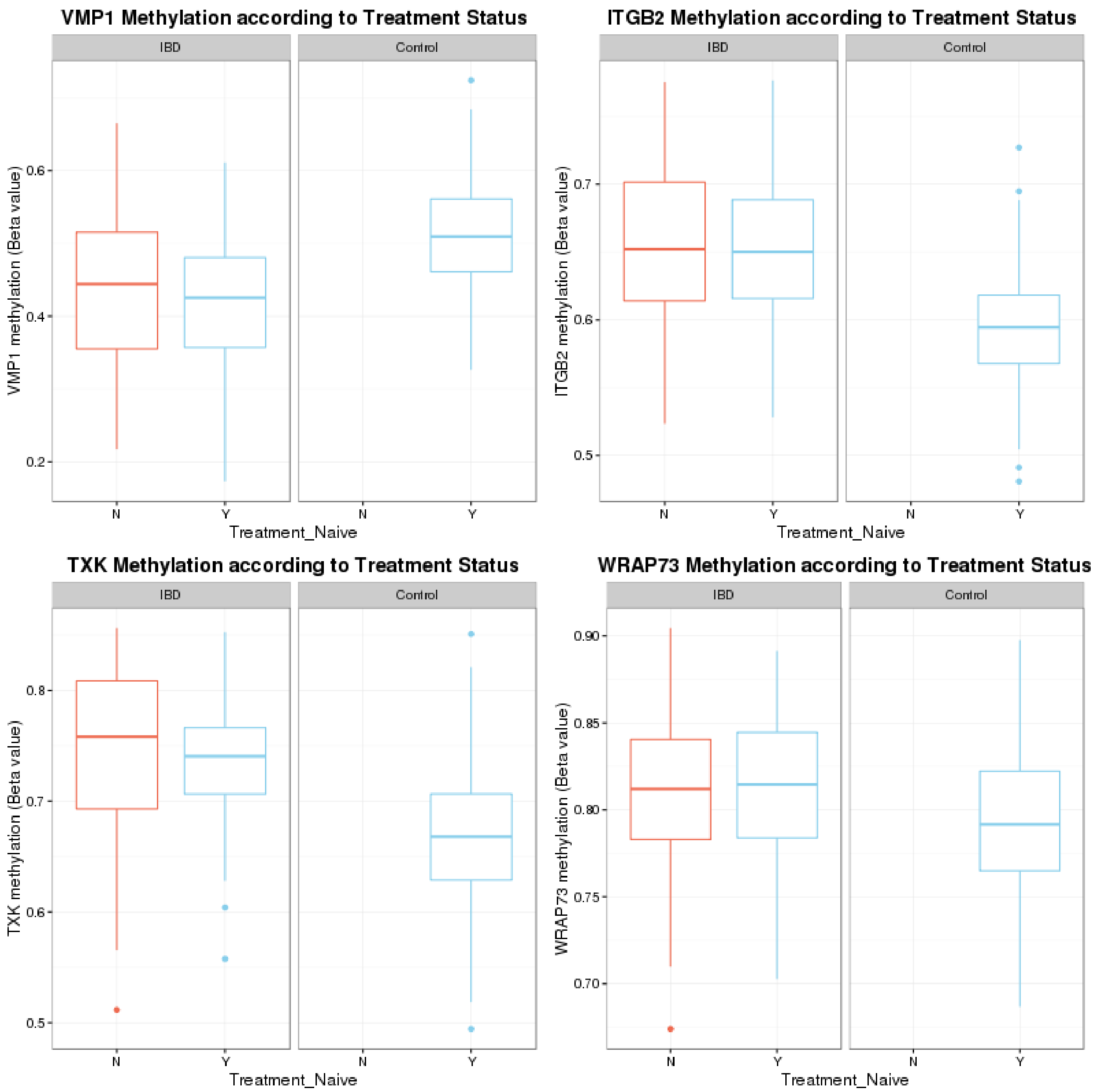


*Supplementary Figure 5 - Boxplot demonstrating the impact of treatment on methylation of top differentially methylated regions (DMRs). y axis denotes methylation beta value for each DMR, and x axis denotes treatment naivety(N=not treatment naïve, y=yes treatment naïve). Facets denote case status IBD= inflammatory bowel disease, Control)*

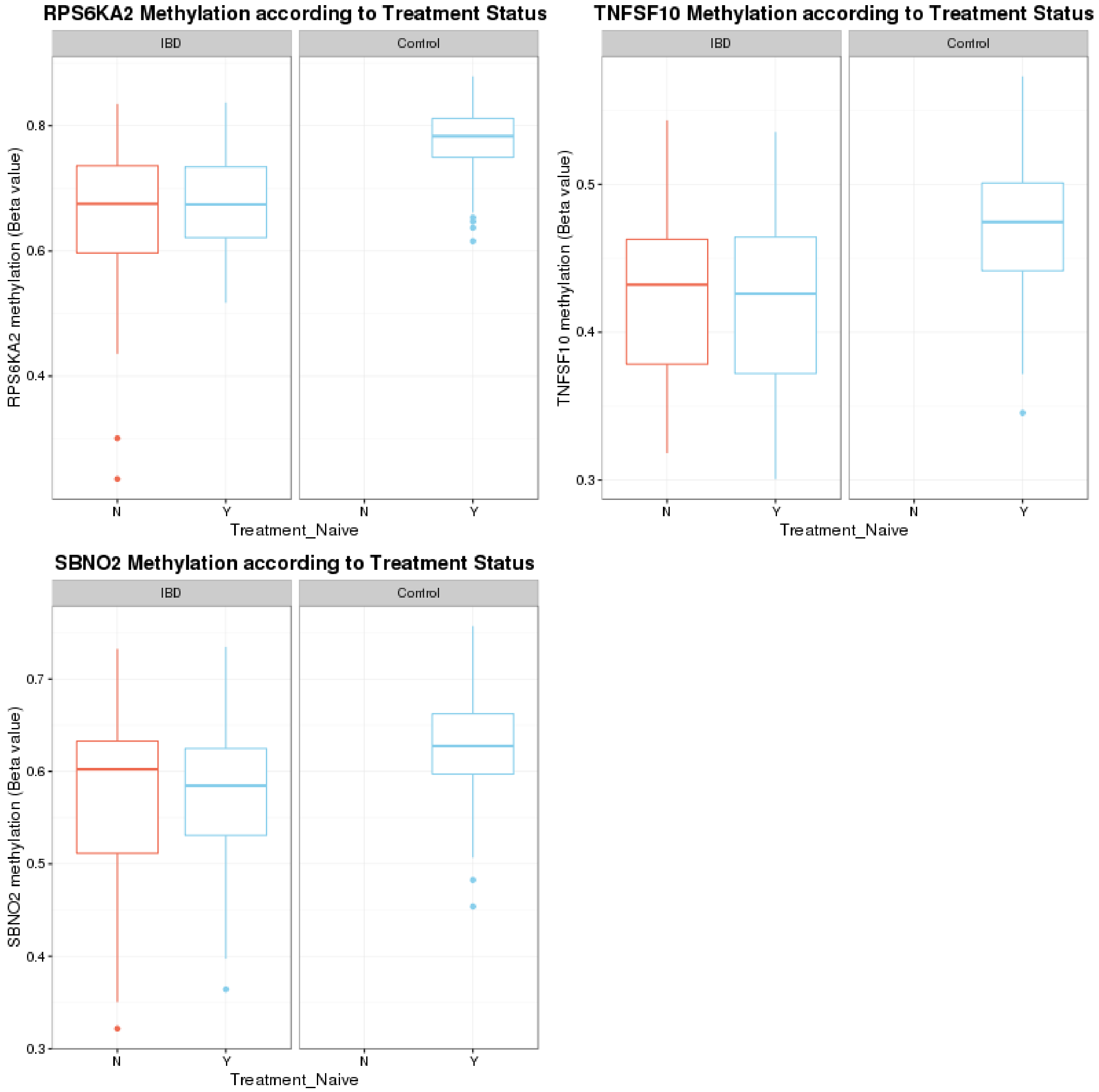


*Supplementary Figure 6 - Boxplot demonstrating the impact of treatment on methylation of top differentially methylated positions (DMPs). y axis denotes methylation beta value for each DMR, and x axis denotes treatment naivety(N=not treatment naïve, y=yes treatment naïve). Facets denote case status IBD= inflammatory bowel disease, Control)*

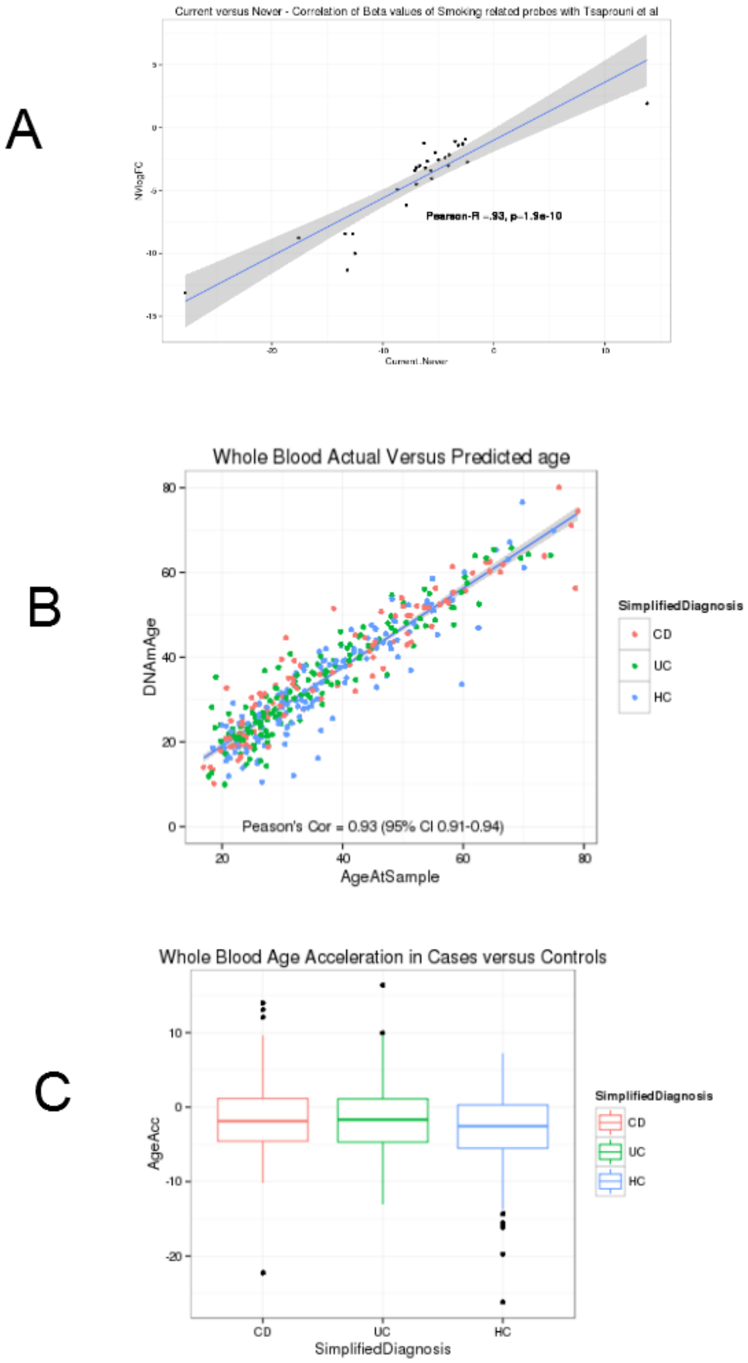


*Supplementary Figure 7 - A - Correlation between smoking related DNA methylation probes in the present dataset (Smokers versus non-smokers) and the previously published dataset by Taylor/Tsapouroni et al.[2] Correlation and p values denotes Pearson's test. B - Correlation between actual age of whole blood samples obtained from patients in this study and estimated age using a modification of the Horvath method[3] Correlation and p values denotes Pearson's test. C – Estimated age acceleration in IBD cases compared with controls using a modification of the Horvath method[3] (CD= Crohn's disease, UC = ulcerative colitis, HC = healthy controls).Y-axis=age acceleration.*

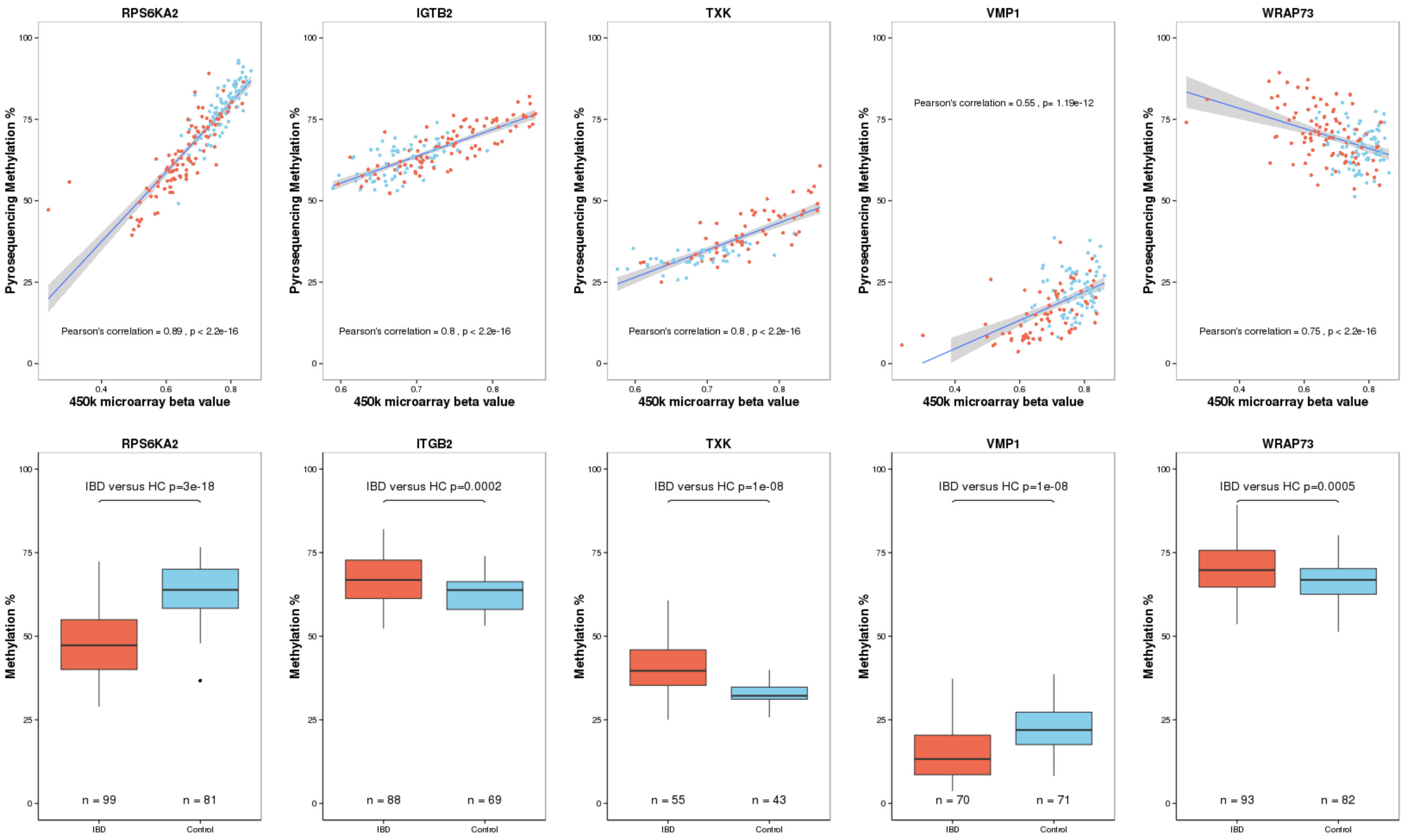


*Supplementary Figure 8 - Technical validation of 450k microarray results using pyrosequencing. The top panel demonstrates correlation between methylation percentage (pyrosequencing) and beta values (450k microarray). The red points represent IBD cases, and the blue points represent controls. The bottom panel demonstrates the methylation difference (%) between IBD cases (red) and controls (blue) using pyrosequencing. P values calculated using Wilcoxon test. Correlation using Pearson's test.*

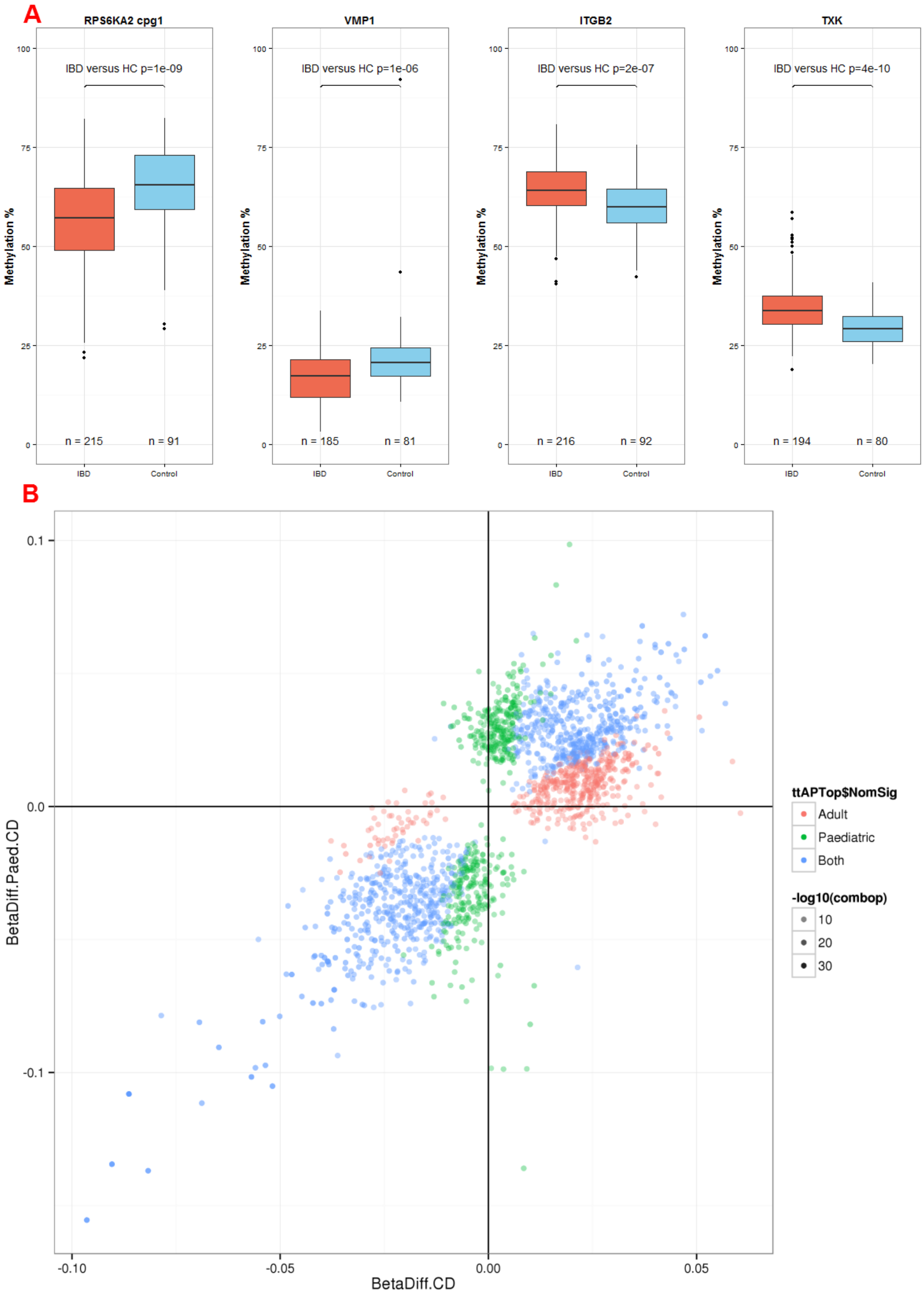


*Supplementary Figure 9 – A= Pyrosequencing validation of top DMP (RPS6KA2) and DMRs (VMP1, ITGB2, TXK) in inflammatory bowel disease cases (IBD, red) and controls (blue). P values calculated using Wilcoxon test. B= Correlation between differentially methylated positions in the present (adult) dataset and previously published paediatric data set. Blue points = methylation probes rank in top 10000 in both adult and paediatric datasets, Red points = methylation probes rank in top 10000 in adult dataset only, Green points = methylation probes rank in top 10000 in paediatric dataset only. Transparency of points denotes the statistical significance (-log10 p value). Pearson's correlation 0.28, p-value < $2.2\times10^{16}$.*

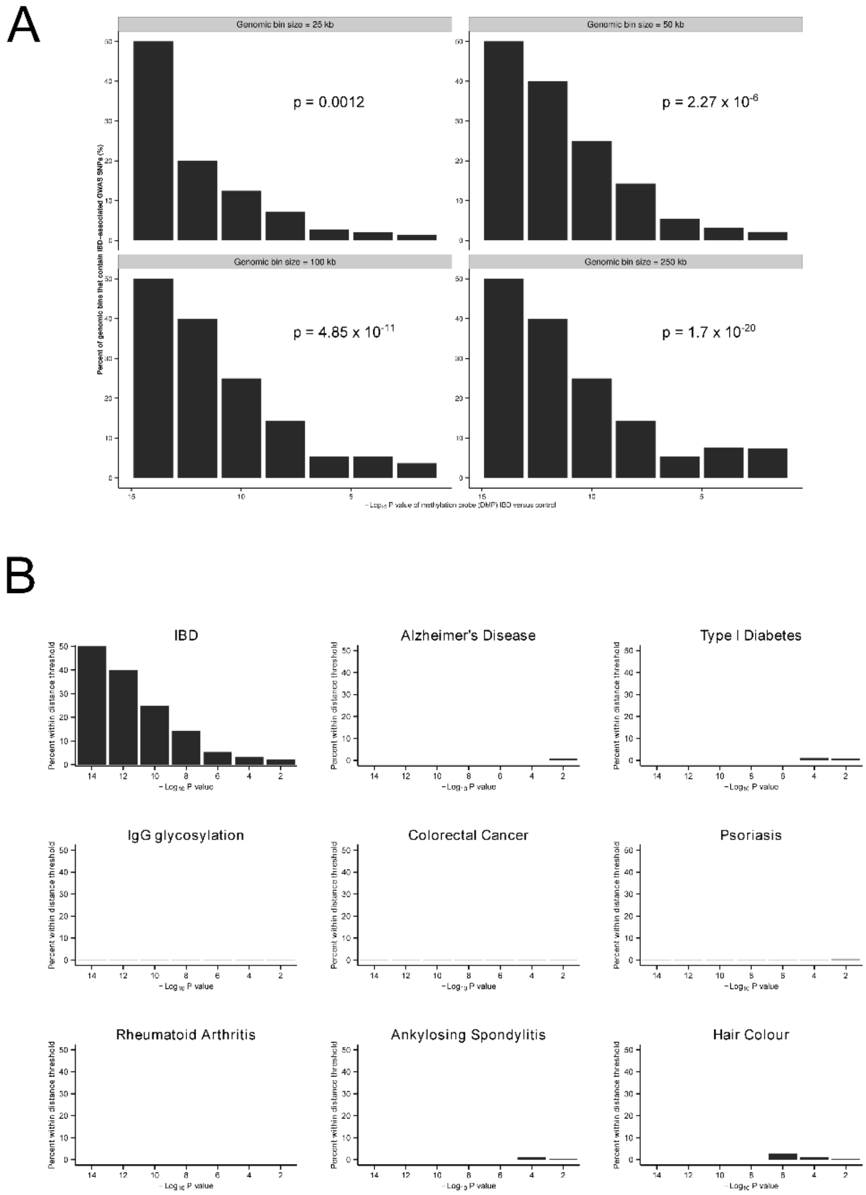


*Supplementary Figure 10- Co-localisation of statistically significant IBD-associated differentially methylated positions (DMP) and IBD GWAS loci. A -Each facet represents a different genomic bin size (25kb, 50kb, 100kb and 250kb). There was co-localisation of IBD-associated SNPs (y-axis) within genomic regions (i.e. defined bin) containing highly statistically significant DNA methylation probes compared those regions containing methylation probes with lower levels of statistical significance (x axis, -log 10 p value). P values denote Wilcoxon rank sum comparison with randomly generated bins with similar probe density. B – Enrichment of IBD-associated SNPs but not of SNPs associated with other complex immune traits and diseases within 50kb regions containing IBD-associated differentially methylated positions.*

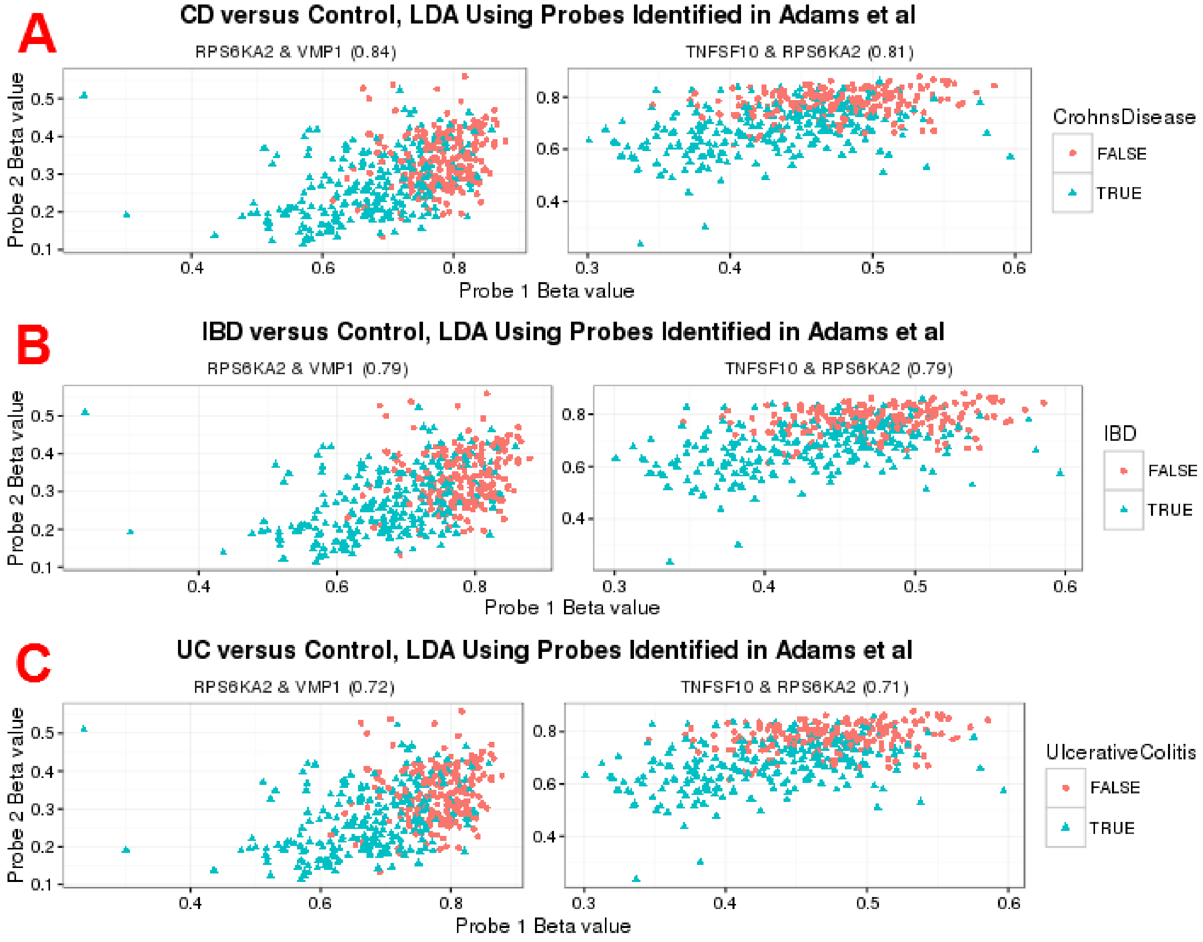


*Supplementary Figure 11 - Validation of 2 methylation probe biomarkers described in Adams et al.[4] The top two performing 2-marker probe sets were validated in the present dataset using linear discriminant analysis (without cross validation) for Crohns disease (CD, blue points, panel A), inflammatory bowel disease (IBD blue points, panel C) and ulcerative colitis (UC blue points, panel C) versus control (red points). The figures in brackets denote the area under receiver operator curve (AUC).*

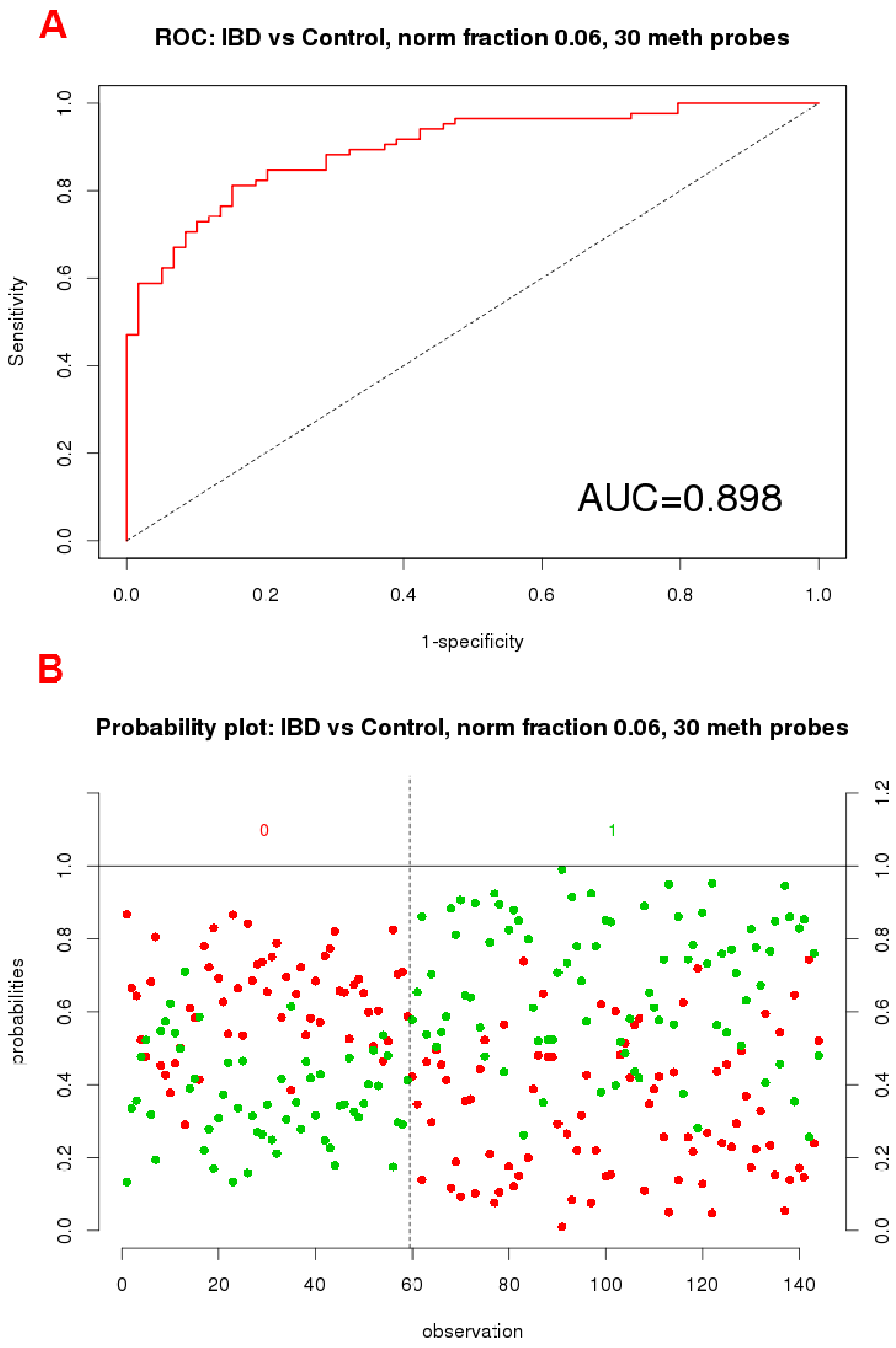


*Supplementary Figure 12 – A: Receiver operating characteristic (ROC) curve for 30 methylation probes selected using L1 penalized regression analysis (Lasso) distinguish IBD from controls. B: Probability plot. 0/red = controls, 1/green = IBD cases.* `

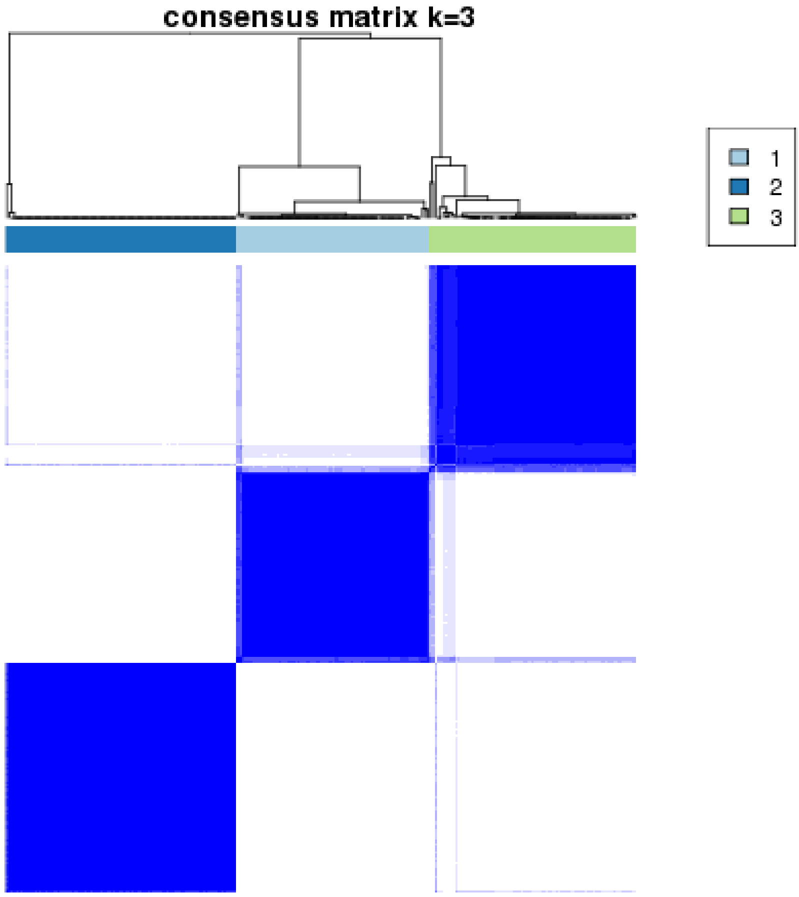


*Supplementary Figure 13- Unsupervised Consensus Clustering to identify IBD subclasses based on DNA methylation data. Three stable clusters were formed.*

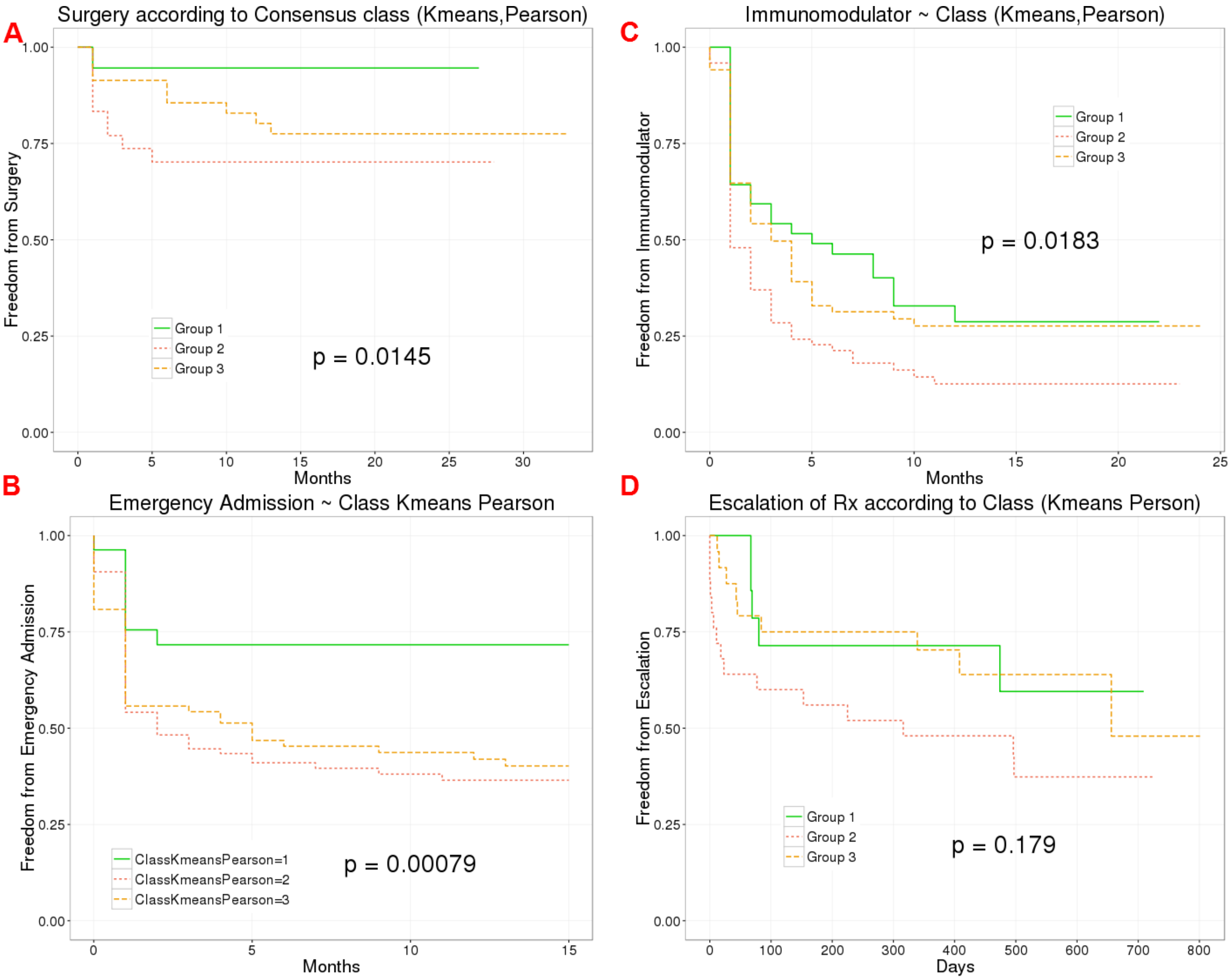


*Supplementary Figure 14 - Survival analysis according to DNA methylation consensus class. A– Time to surgery in months. B – Time until emergency admission in months. C – Time until requirement for Immunomodulator in months (oral or IV steroid, anti-TNFalpha drug, ciclosporin, Methotrexate, thiopurine). D – Criteria of treatment escalation defined by Lee et al[5] in days (surgery, step up to 2 or more immunmodulators). P values denote ChiSquared test for difference between survival curves with 2 degrees of freedom. The coloured lines denoted the three different groups (green =Group 1, low risk, yellow = Group 3, intermediate risk, red= Group 2 high risk).*

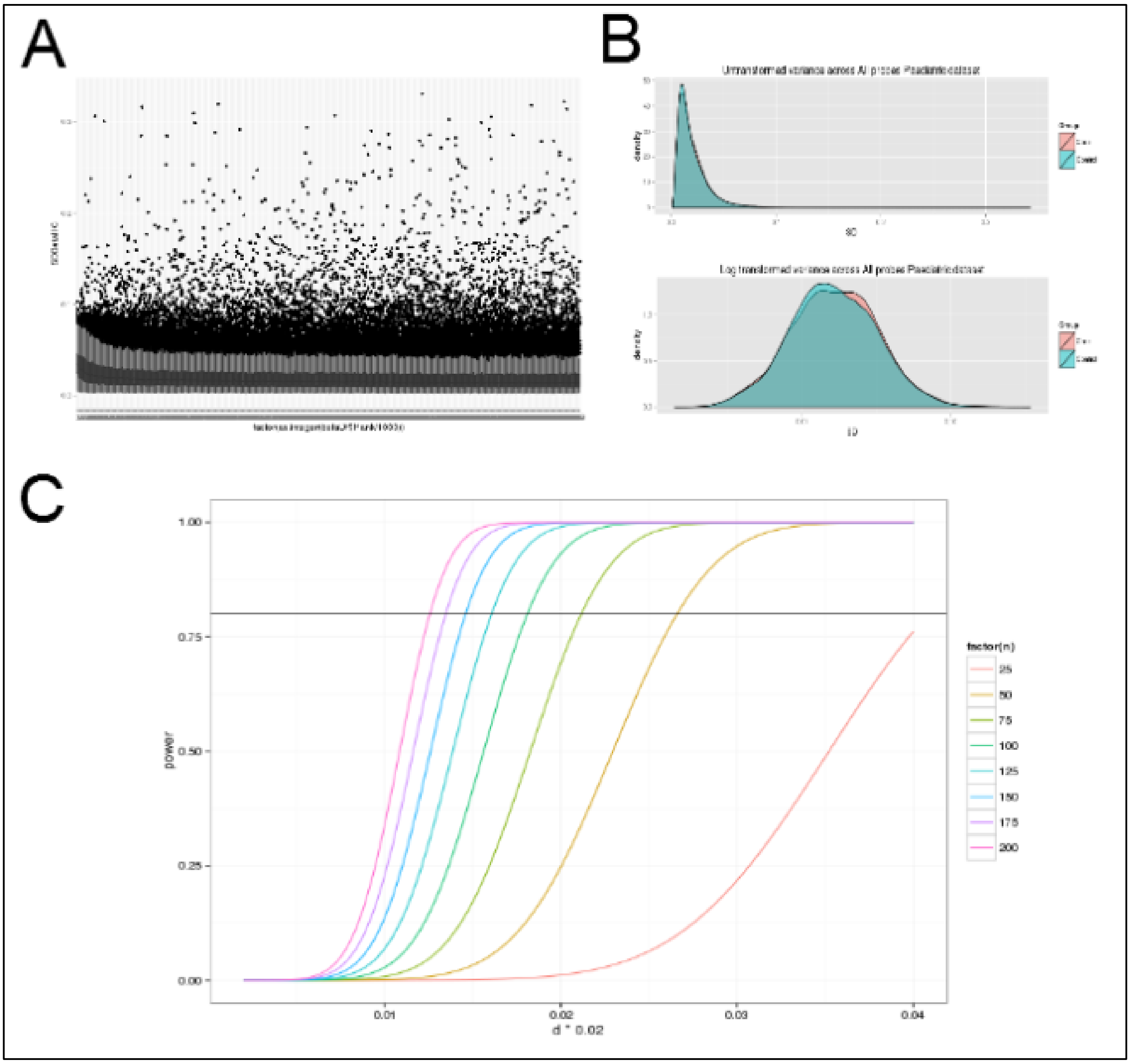


*Supplementary Figure 15 – Power A – Boxplot of median variance across all probes on 450K array. B – Distribution of beta values and log transformed beta values. C – Power curves used to determine numbers per group required to detect a difference of one standard deviation. Y axis = power, X axis = effect size, different colour lines represent number (n) in each group.*

| | | **CD (n=121)** | **UC (n=119)** | P value CD vs UC | **Symptomatic controls (n=74)** | P value SC vs CD | P value SC vs UC | **Healthy volunteers (n=117)** | P value HL vs CD | P value HL vs UC |
|---|---|---|---|---|---|---|---|---|---|---|
| **Age** [median(IQR)] | | 32.4 (24.9-50.7) | 34.3 (25.5-47.8) | 1† | 32.8 (26.4-45.5) | 0.9† | 0.9† | 32.3 26.4-40.6) | 0.4† | 0.3† |
| **Females** (%) | | 58 (47.9) | 51 (42.9) | 0.5 ◊ | 39 (52.7) | 0.6 ◊ | 0.2 ◊ | 59 (50.4) | 0.8◊ | 0.3◊ |
| **Smoking status** | Current | 53 | 13 | Current versus ex/never $5\times10^{-8}$ | 17 | Current versus ex/never 0.005 | Current versus ex/never 0.05 | 24 | Current versus ex/never 0.0005 | Current versus ex/never 0.5 |
| | Ex | 29 | 45 | | 17 | | | 32 | | |
| | Never | 39 | 58 | | 40 | | | 56 | | |
| | Unknown | 0 | 3 | | 0 | | | 5 | | |
| **CRP** | | 8(2-23) | 11.5 (2-31) | 0.8† | 0 (0-3.5) | 0.006† | 0.004† | | | |
| **ESR** | | 18 (5-39) | 5.5 (4.3-9.8) | 0.03† | 6 (4.5-7.5) | 0.002† | 1† | | | |
| **FC** | | 495 (135-828) | 760 (660-950) | 0.2 | 19 (19-37) | 0.0001† | $1.0\times10^{-6}$† | | | |
| **Time between diagnosis & sample (days, median [IQR])** | | 48 [7-90.8) | 32 [1-71] | | | | | | | |
| **Treatment Naïve (denominator number with available data)** | | 27/69 | 37/70 | | | | | | | |
| **Oral/IV steroids at sample (duration of therapy days, median [IQR])** | | 10/69 (8[2-14]) | 10/70 (2[1-6.5]) | | | | | | | |
| **Biologic at sample (duration of therapy in days, median [IQR])** | | 4/69 (4 [2.75-5.25]) | - | | | | | | | |
| **Aza/6MP at sample (duration of therapy in days, median [IQR])** | | 10/69 (5[2-10]) | 4/70 (5[2-10]) | | | | | | | |

| | | | | | | |
|---|---|---|---|---|---|---|
| **Topical therapy at sample (duration of therapy in days, median [IQR])** | 1/69 (1 DAY) | 11/70 (5[2.75-13.25]) | | | | |
| **Oral 5ASA at sample (duration of therapy in days, median [IQR])** | 3/69 (2[1-5]) | 16/70 (2[1-5) | | | | |

*Supplementary Table 1 - Patient Demographics (CD= Crohn's disease, UC= Ulcerative colitis, SC= Symptomatic controls, HL=Healthy Lab volunteers, IQR=interquartile range, CRP=C-reactive protein, ESR=Erythrocyte sedimentation rate, FC= faecal calprotectin † = Wilcoxon rank sum test, ǂ = Welch two sample t test, ◊ = $\chi^2$ test)*

| category | No. genes differentially expressed in category | Total No of genes in category | GO term | P Val | FDR Adj P Val |
|---|---|---|---|---|---|
| GO:0080134 | 47 | 1293 | regulation of response to stress | 1.27E-07 | 0.003 |
| GO:0048518 | 119 | 4815 | positive regulation of biological process | 3.41E-07 | 0.003 |
| GO:0044763 | 213 | 10900 | single-organism cellular process | 8.02E-07 | 0.005 |
| GO:0044699 | 227 | 11972 | single-organism process | 1.02E-06 | 0.005 |
| GO:0009607 | 32 | 816 | response to biotic stimulus | 1.61E-06 | 0.005 |
| GO:0001775 | 34 | 853 | cell activation | 1.70E-06 | 0.005 |
| GO:0002252 | 29 | 675 | immune effector process | 1.93E-06 | 0.005 |
| GO:0048583 | 86 | 3212 | regulation of response to stimulus | 2.38E-06 | 0.005 |
| GO:0048522 | 104 | 4164 | positive regulation of cellular process | 2.50E-06 | 0.005 |
| GO:0051179 | 120 | 5016 | localization | 2.59E-06 | 0.005 |
| GO:0031347 | 29 | 699 | regulation of defense response | 2.80E-06 | 0.005 |
| GO:0002376 | 66 | 2336 | immune system process | 3.56E-06 | 0.006 |
| GO:0043207 | 30 | 783 | response to external biotic stimulus | 5.01E-06 | 0.007 |
| GO:0051707 | 30 | 783 | response to other organism | 5.01E-06 | 0.007 |
| GO:0002366 | 13 | 171 | leukocyte activation in immune response | 5.27E-06 | 0.007 |
| GO:0045321 | 27 | 635 | leukocyte activation | 5.86E-06 | 0.007 |
| GO:0002263 | 13 | 173 | cell activation involved in immune response | 6.12E-06 | 0.007 |
| GO:0002682 | 44 | 1365 | regulation of immune system process | 1.11E-05 | 0.012 |

| | | | | | |
|---|---|---|---|---|---|
| GO:0006810 | 99 | 4044 | transport | 1.23E-05 | 0.013 |
| GO:0051234 | 101 | 4156 | establishment of localization | 1.45E-05 | 0.014 |
| GO:0070887 | 67 | 2444 | cellular response to chemical stimulus | 1.81E-05 | 0.017 |
| GO:0006897 | 24 | 546 | endocytosis | 2.86E-05 | 0.026 |
| GO:0030099 | 17 | 316 | myeloid cell differentiation | 3.18E-05 | 0.027 |
| GO:0006952 | 47 | 1610 | defense response | 3.27E-05 | 0.027 |
| GO:0045638 | 8 | 77 | negative regulation of myeloid cell differentiation | 3.93E-05 | 0.031 |
| GO:0051049 | 48 | 1584 | regulation of transport | 4.45E-05 | 0.034 |
| GO:0046632 | 8 | 75 | alpha-beta T cell differentiation | 4.72E-05 | 0.035 |
| GO:0009891 | 49 | 1645 | positive regulation of biosynthetic process | 5.25E-05 | 0.037 |
| GO:0009611 | 33 | 950 | response to wounding | 6.13E-05 | 0.040 |
| GO:0010646 | 71 | 2666 | regulation of cell communication | 6.27E-05 | 0.040 |
| GO:0050776 | 31 | 885 | regulation of immune response | 6.40E-05 | 0.040 |
| GO:0002684 | 29 | 818 | positive regulation of immune system process | 6.41E-05 | 0.040 |
| GO:0044422 | 150 | 7205 | organelle part | 7.51E-05 | 0.042 |
| GO:1902578 | 89 | 3663 | single-organism localization | 7.54E-05 | 0.042 |
| GO:0002521 | 19 | 413 | leukocyte differentiation | 7.56E-05 | 0.042 |
| GO:0044765 | 86 | 3512 | single-organism transport | 7.67E-05 | 0.042 |
| GO:0051641 | 69 | 2638 | cellular localization | 7.72E-05 | 0.042 |
| GO:0046637 | 6 | 43 | regulation of alpha-beta T cell differentiation | 8.18E-05 | 0.042 |

| | | | | | |
|---|---|---|---|---|---|
| GO:004853 4 | 27 | 706 | hematopoietic or lymphoid organ development | 8.27E-05 | 0.042 |
| GO:001619 2 | 39 | 1186 | vesicle-mediated transport | 8.42E-05 | 0.042 |
| GO:005089 6 | 152 | 7392 | response to stimulus | 8.59E-05 | 0.042 |
| GO:004877 1 | 10 | 132 | tissue remodeling | 8.89E-05 | 0.042 |
| GO:003336 5 | 27 | 744 | protein localization to organelle | 9.73E-05 | 0.044 |
| GO:005077 8 | 23 | 587 | positive regulation of immune response | 9.78E-05 | 0.044 |
| GO:000227 4 | 10 | 143 | myeloid leukocyte activation | 0.00010 3 | 0.046 |
| GO:000960 5 | 61 | 2303 | response to external stimulus | 0.00010 8 | 0.046 |
| GO:004508 7 | 32 | 974 | innate immune response | 0.00010 9 | 0.046 |
| GO:000989 3 | 80 | 3185 | positive regulation of metabolic process | 0.00011 1 | 0.046 |
| GO:000269 7 | 17 | 371 | regulation of immune effector process | 0.00011 5 | 0.046 |
| GO:000695 5 | 42 | 1454 | immune response | 0.00011 7 | 0.046 |
| GO:002305 1 | 69 | 2620 | regulation of signaling | 0.00012 | 0.046 |
| GO:003210 1 | 27 | 763 | regulation of response to external stimulus | 0.00012 | 0.046 |
| GO:190370 7 | 9 | 119 | negative regulation of hemopoiesis | 0.00012 3 | 0.046 |
| GO:000695 0 | 85 | 3582 | response to stress | 0.00012 3 | 0.046 |

*Supplementary Table 2 – GO term analysis of DMPs in IBD versus control in whole blood*

| Gene | Position | CHR | Δβ | P Value | Holm adj P value |
|---|---|---|---|---|---|
| RPS6KA2 | 166970252 | chr6 | -0.09 | 1.26E-29 | 5.63E-24 |
| SBNO2 | 1130866 | chr19 | -0.09 | 3.61E-26 | 1.62E-20 |
| VMP1 | 57915665 | chr17 | -0.11 | 3.04E-23 | 1.36E-17 |
| SBNO2 | 1130965 | chr19 | -0.05 | 1.15E-22 | 5.16E-17 |
| VMP1 | 57915717 | chr17 | -0.10 | 3.52E-22 | 1.58E-16 |
| NA | 50327986 | chr22 | -0.05 | 4.81E-21 | 2.15E-15 |
| NA | 101901234 | chr3 | -0.06 | 2.73E-18 | 1.22E-12 |
| SOCS3 | 76354621 | chr17 | -0.07 | 3.63E-18 | 1.63E-12 |
| NA | 12890029 | chr19 | -0.06 | 1.92E-17 | 8.62E-12 |
| FKBP5 | 35654363 | chr6 | -0.07 | 3.42E-17 | 1.53E-11 |
| NA | 35696870 | chr6 | -0.04 | 7.67E-17 | 3.44E-11 |
| VMP1 | 57915773 | chr17 | -0.06 | 1.09E-16 | 4.90E-11 |

*Supplementary Table 3 - Top table of differentially methylated positions (DMPs) between Crohn's disease (CD) cases and controls in whole blood. NA denotes methylation probes with no annotated gene symbol. Δβ = difference in methylation beta value. Holm adjusted p value derived from linear models with age, sex and estimated cell proportions as co-variates. Chr= chromosome.*

| Gene | Position | CHR | Δβ | P Value | Holm adj P value |
|---|---|---|---|---|---|
| NA | 35696870 | chr6 | -0.04 | 1.12E-16 | 5.02E-11 |
| NA | 101901234 | chr3 | -0.06 | 1.35E-16 | 6.07E-11 |
| TNFSF10 | 172235808 | chr3 | -0.05 | 2.85E-16 | 1.28E-10 |
| SBNO2 | 1130866 | chr19 | -0.06 | 1.42E-15 | 6.36E-10 |
| NA | 12890029 | chr19 | -0.06 | 1.57E-15 | 7.03E-10 |
| AIM2 | 159047163 | chr1 | -0.06 | 6.96E-15 | 3.12E-09 |
| ICA1 | 8201134 | chr7 | 0.04 | 1.45E-14 | 6.48E-09 |
| RPS6KA2 | 166970252 | chr6 | -0.07 | 2.22E-14 | 9.95E-09 |
| VMP1 | 57915665 | chr17 | -0.09 | 2.48E-14 | 1.11E-08 |
| ZEB2 | 145172035 | chr2 | -0.08 | 5.65E-14 | 2.53E-08 |
| NA | 50327986 | chr22 | -0.04 | 2.11E-13 | 9.45E-08 |
| FRMD4A | 13913931 | chr10 | 0.05 | 2.45E-13 | 1.10E-07 |

*Supplementary Table 4 - Top table of differentially methylated positions (DMPs) between Ulcerative colitis (UC) cases and controls in whole blood. NA denotes methylation probes with no annotated gene symbol. Δβ = difference in methylation beta value. Holm adjusted p value derived from linear models with age, sex and estimated cell proportions as co-variates. Chr= chromosome.*

| Gene | Probe | Position | CHR | Δβ | P Value | Holm Adj P value |
|---|---|---|---|---|---|---|
| MNDA | cg05304729 | 158800024 | chr1 | -0.06 | 3.44E-07 | 0.15 |
| NA | cg19683494 | 74908142 | chr5 | -0.06 | 5.94E-07 | 0.27 |
| ZEB2 | cg10502206 | 145182344 | chr2 | 0.02 | 1.08E-06 | 0.49 |
| NA | cg02573091 | 74908125 | chr5 | -0.07 | 1.97E-06 | 0.88 |
| NLRC5 | cg07839457 | 57023022 | chr16 | -0.06 | 2.81E-06 | 1 |
| TK1 | cg25069807 | 76171191 | chr17 | 0.03 | 4.75E-06 | 1 |
| NA | cg25730685 | 2375010 | chr1 | 0.01 | 7.07E-06 | 1 |
| SPG7 | cg04879696 | 89574810 | chr16 | 0.01 | 7.66E-06 | 1 |
| TK1 | cg06098276 | 76171208 | chr17 | 0.04 | 1.30E-05 | 1 |
| CENPV | cg05238069 | 16257135 | chr17 | 0.01 | 1.56E-05 | 1 |
| CAPN5 | cg08103551 | 76777993 | chr11 | 0.00 | 1.67E-05 | 1 |
| ADK | cg23198334 | 76179907 | chr10 | 0.01 | 2.10E-05 | 1 |

*Supplementary Table 5 - Top table of differentially methylated positions (DMPs) between Crohn's disease (CD) and Ulcerative colitis (UC) in whole blood. NA denotes methylation probes with no annotated gene symbol. Δβ = difference in methylation beta value. Holm adjusted p value derived from linear models with age, sex and estimated cell proportions as co-variates. Chr= chromosome.*

| Gene | Probe | Position | CHR | Δβ | P Value | Holm adj P value |
|---|---|---|---|---|---|---|
| ROCK1 | cg09449490 | 18690843 | chr18 | 0.01 | 1.17E-06 | 0.52 |
| PFKFB3 | cg27545615 | 6249748 | chr10 | 0.03 | 1.55E-06 | 0.69 |
| PIK3R6 | cg00409104 | 8762014 | chr17 | 0.02 | 1.55E-06 | 0.70 |
| CELF2 | cg11832281 | 11211022 | chr10 | 0.01 | 1.83E-06 | 0.82 |
| NA | cg01686975 | 138816336 | chr7 | 0.02 | 2.19E-06 | 0.98 |
| NA | cg24448340 | 179921042 | chr1 | 0.03 | 3.00E-06 | 1 |
| TXNDC11 | cg03382501 | 11794641 | chr16 | 0.02 | 3.06E-06 | 1 |
| EFHD2 | cg25978218 | 15738732 | chr1 | 0.02 | 3.08E-06 | 1 |
| CPD | cg23344321 | 28707212 | chr17 | 0.02 | 4.57E-06 | 1 |
| XPO6 | cg26730763 | 28205389 | chr16 | 0.01 | 5.56E-06 | 1 |
| KIAA1033 | cg13622209 | 105501127 | chr12 | 0.01 | 5.75E-06 | 1 |
| NA | cg08900384 | 72546168 | chr11 | 0.02 | 6.03E-06 | 1 |

*Supplementary Table 6 - Top table of differentially methylated positions (DMPs) between Symptomatic controls and healthy volunteers in whole blood. NA denotes methylation probes with no annotated gene symbol. Δβ = difference in methylation beta value. Holm adjusted p value derived from linear models with age, sex and estimated cell proportions as co-variates. Chr= chromosome.*

| Differentially methylated regions (DMRs) | |
|---|---|
| **VMP1/ microRNA-21** | Vaculole membrane protein1 (VMP1) encodes a transmembrane protein within the golgi body, endoplastic reticulum.[6] VMP1 is an inducer of autophagy via interactions with BECN1.[7,8]<br><br>microRNA-21, is a microRNA is encoded at the 3' end of VMP1,[9] where the change in methylation is concentrated at in IBD.[4] microRNA-21 has been linked with several types of cancer including colorectal cancer.[10] microRNA-21 has been widely implicated in pathogenesis of IBD.[11] microRNA-21 knockout mice have improve survival following chemically (Dextran sulphate sodium) induced colitis in two studies,[12] but exacerbation in TBNS (2,4,6-trinitrobenzenesulfonic acid) and T-cell transfer models of murine colitis.[13] Several microRNA screens have identified upregulation of microRNA-21 in IBD, notably in cases with active inflammation.[14–18] |
| **WDR8/WRAP 73** | WD (trp-asp) repeat protein family, antisense to Trp73. The WD gene repeat motif make up a large family of genes involved in several cellular and gene regulatory processes, including cell cycle progression, apoptosis and signal transduction.[19] Murine studies suggest a role for WRAP73 in the process of ossification.[19] |
| **ITGB2 (CD18)** | Integrin Beta 2 subunit. Cell adhesion and cell surface mediated signalling.[20] Heritable defects in this gene cause leukocyte adhesion deficiency type I characterised by severe recurrent infections.[21,22] Aberrant DNA methylation at the ITGB2 locus has previous been demonstrated in IBD[23] and other diseases.[24,25]<br><br>Anti-integrin antibodies have attracted interest as therapeutics in IBD. Natalizumab is an anti-alpha4 integrin antibody is efficacious in multiple sclerosis and CD, but is associated an unacceptable risk of PML (progressive multifocal leukoenchepalopathy).[26] Vedolizumab is a gut-specific anti-α4β7 integrin antibody efficacious for inducing and maintaining remission in UC (GEMINI-1)[27] and CD.[28] |
| **TXK** | TXK is a member of the Tec family of tyrosine kinases.[29] T cells express TXK and the other tec kinases, which serve as modulators of T-cell receptor signalling and assist in cytokine production by CD4+ effector T-cells.[30] TXK may also have role in T-cell development in the thymus. TXK has been shown to be over expressed in the circulating leucocytes in Behcet's disease [31] including in Th1 lymphocytes accumulating within intestinal lesions.[32] An IBD-associated GWAS locus.[33] |
| **HDAC4** | Histone Deacetylase class II. Histone modifications are an important epigenetic mechanism that are associated with alternative conformations of chromatin. Acetylation of lysine on histones H3 and H4 is associated with transcriptional activity.[34] The extent of acetylation are regulated by the relative activity of the HDAC enzymes, together with the HAT (histone acetyl transferases).[34] Non-specific HDAC inhibitors such as butyrate are associated with amelioration of colitis.[35] Alternative methylation of HDAC4 has previously been described in other situations.[25,36,37] |
| **Differentially methylated positions (DMPs)** | |
| **RPS6KA2** | Ribsomal S6 kinase A2 is a ribosomal kinase in the serine/threonine kinase family.[38] Acts on the intracellular MAP |

| | |
|---|---|
| **(RSK3)** | kinase signaling pathway (interacts with MAPK1 and 3).[39] Thought to have a role in cell growth, cell motility, proliferation[39] and cell cycle progression,[40] although may also act as a tumour suppressor gene in ovarian cancer.[41] Alternatively spliced isoforms exist.[41] An IBD-associated GWAS locus.[33] |
| **SBNO2** | Strawberry notch homologue 2 has an anti-inflammatory effect, by acting in the IL-10 downstream pathway.[42] IL-10 induced SNBNO2 expression was found to repress NF-κβ (but not IRF7) selectively within macrophages.[42] A susceptibility locus in IBD GWAS.[43] |
| **TNFSF10 (TRAIL, CD253, Apo2L)** | Tumour necrosis factor superfamily member 10/TRAIL acts as a ligand in the TNF family.[44] This widely investigated cytokine induces caspase-8-dependent apoptosis in tumour but not normal cells;[45] this property has led to extensive investigation as a chemotherapeutic agent.[46] TRAIL has been implicated in intestinal inflammation and demonstrated to be over-expressed in intestinal epithelial cell lines[47] and in human mononuclear cells in inflamed intestinal sections.[48] |
| **BCL3** | B-cell CLL/lymphoma 3 is a proto-oncogene acting as a co-activator through NF-κβ and is associated with translocation in a specific form of B-cell leaukaemia (t(14;19)(q32;q13)) [49] |
| **IL23A** | Interleukin 23 subunit A. The T-helper (Th)17 and interleukin (IL)12-23 pathway is well established in IBD pathogenesis, with susceptibility gene loci IL23R, IL12B, JAK2, and STAT3 identified in both UC and CD.[50,51] |

*Supplementary Table 7 - Selected candidate genes in differentially methylated regions and positions.*

| Illumina 450k Probe id | Chr | Gene symbol | Δβ | P.Value | FDR adj.P.Val |
|---|---|---|---|---|---|
| cg17501210 | chr6 | RPS6KA2 | -0.07 | 2.09E-21 | 9.38E-16 |
| cg18608055 | chr19 | SBNO2 | -0.07 | 2.20E-20 | 9.84E-15 |
| cg16936953 | chr17 | VMP1 | -0.08 | 1.20E-18 | 5.36E-13 |
| cg09349128 | chr22 | NA | -0.04 | 6.81E-18 | 3.05E-12 |
| cg12170787 | chr19 | SBNO2 | -0.04 | 6.96E-18 | 3.12E-12 |
| cg25114611 | chr6 | NA | -0.04 | 1.19E-17 | 5.35E-12 |
| cg12992827 | chr3 | NA | -0.05 | 3.58E-17 | 1.60E-11 |
| cg19821297 | chr19 | NA | -0.06 | 1.79E-16 | 8.04E-11 |
| cg12054453 | chr17 | VMP1 | -0.07 | 4.29E-16 | 1.92E-10 |
| cg01059398 | chr3 | TNFSF10 | -0.04 | 1.78E-15 | 7.98E-10 |
| cg26804423 | chr7 | ICA1 | 0.04 | 4.04E-15 | 1.81E-09 |
| cg02716826 | chr9 | NA | -0.03 | 8.06E-15 | 3.61E-09 |
| cg13619623 | chr7 | BBS9 | 0.04 | 1.00E-14 | 4.49E-09 |
| cg18942579 | chr17 | VMP1 | -0.05 | 1.05E-14 | 4.70E-09 |
| cg03546163 | chr6 | FKBP5 | -0.06 | 1.72E-14 | 7.70E-09 |
| cg07398517 | chr3 | NA | -0.03 | 1.95E-14 | 8.73E-09 |
| cg26470501 | chr19 | BCL3 | -0.03 | 2.19E-14 | 9.82E-09 |
| cg22959742 | chr10 | FRMD4A | 0.04 | 5.91E-14 | 2.65E-08 |
| cg02448796 | chr1 | KCNAB2 | 0.04 | 1.15E-13 | 5.14E-08 |
| cg16724148 | chr1 | AGL | 0.03 | 3.11E-13 | 1.39E-07 |

*Supplementary Table 8 - Top table of differentially methylated positions (DMPs) between inflammatory bowel disease (IBD) cases and controls in whole blood with smoking included as a covariate along with age, sex and the estimated blood cell proportions. NA denotes methylation probes with no annotated gene symbol. Δβ = difference in methylation beta value. FDR = false discovery rate p value. Chr= chromosome.*

| Illumina 450k Probe id | Chr | Gene symbol | Δβ | P.Value | Holm adj.P.Val |
|---|---|---|---|---|---|
| cg16936953 | chr17 | VMP1 | -0.11 | 1.71E-16 | 7.68E-11 |
| cg01059398 | chr3 | TNFSF10 | -0.06 | 2.83E-16 | 1.27E-10 |
| cg18608055 | chr19 | SBNO2 | -0.08 | 3.65E-16 | 1.64E-10 |
| cg19821297 | chr19 | NA | -0.07 | 9.37E-15 | 4.20E-09 |
| cg09349128 | chr22 | NA | -0.05 | 2.09E-14 | 9.37E-09 |
| cg12054453 | chr17 | VMP1 | -0.09 | 6.02E-14 | 2.70E-08 |
| cg12992827 | chr3 | NA | -0.06 | 3.00E-13 | 1.35E-07 |
| cg12269535 | chr6 | SRF | -0.05 | 8.07E-13 | 3.62E-07 |
| cg12170787 | chr19 | SBNO2 | -0.05 | 1.43E-12 | 6.41E-07 |
| cg02650017 | chr17 | PHOSPHO1 | -0.02 | 2.44E-12 | 1.09E-06 |
| cg16292768 | chr8 | CLU | -0.05 | 3.22E-12 | 1.44E-06 |
| cg17501210 | chr6 | RPS6KA2 | -0.07 | 3.80E-12 | 1.70E-06 |
| cg02716826 | chr9 | NA | -0.04 | 9.09E-12 | 4.07E-06 |
| cg10472711 | chr7 | HEATR2 | -0.06 | 2.62E-11 | 1.17E-05 |

*Supplementary Table 9 - Top table of differentially methylated positions (DMPs) between inflammatory bowel disease (IBD) cases and controls in whole blood with treatment status included as a covariate along with age, sex and the estimated blood cell proportions. NA denotes methylation probes with no annotated gene symbol. Δβ = difference in methylation beta value.*

| | **IBD** (n=130) | **Control** (n=101) | p Value |
|---|---|---|---|
| **Female** | 51 (50.5%) | 47 (36.2%) | 0.3◊ |
| **Age** (median, IQR) | 29.2 (25.9-42.4) | 30.7 (26 - 38.9) | 0.9† |
| **Current Smokers** (%) | 36 (27.7%) | 20 (19.8%) | 0.2◊ |
| **CRP** | 8.5 (3-33.5) | 2 (1-4.5) | 1.7e-05† |
| **Albumin** | 34 (26.3-38) | 41 (38-43) | 2.3e-07† |
| **WCC** | 8.3 (5.8-12.3) | 5.7 (4.8-12.3) | 6.1e-07† |

*Supplementary Table 10 - Patient demographics of subset of adult cohort used for technical validation studies using pyrosequencing (Results are median and interquartile range unless stated, WCC=white cell count, CRP=C-reactive protein, IQR= interquartile range, IBD=inflammatory bowel disease, † = Wilcoxon rank sum test, ◊ = $\chi 2$ test)*

| | Pearson's correlation | Lower CI | Upper CI | P value |
|---|---|---|---|---|
| **RPS6KA2** | 0.89 | 0.86 | 0.92 | 2.2e-16 |
| **IGTB2** | 0.8 | 0.73 | 0.85 | 2.2e-16 |
| **TXK** | 0.8 | 0.71 | 0.86 | 2.2e-16 |
| **VMP1** | 0.55 | 0.43 | 0.66 | 1.2e-12 |
| **WRAP73** | 0.75 | 0.68 | 0.81 | 2.2e-16 |

*Supplementary Table 11 - Technical replication: Correlation between Methylation 450k microarray and pyrosequencing for same samples. CI = 95% confidence interval*

| | | CD (n=121) | UC (n=119) | Controls (n= 98) |
|---|---|---|---|---|
| **Age** | | 36.5 (26.4-47.8) | 36.6 (27.1-46.7) | 34.9 (27.3-55.5) |
| **Females** *(%)* | | 68 *(57)* | 74 *(62)* | 65 *(66)* |
| **Smoking status** | Current *(%)* | 18 *(15)* | 7 *(6)* | 14 *(14)* |

*Supplementary Table 12 - Patient demographics of Independent pyrosequencing cohort (data presented are medians (interquartile range) except where specified)*

| Rank IBD | Rank CD | Probe ID | Chr | Gene Symbol | Δβ IBD | Holm adj P Value IBD | Δβ CD | Holm adj P Value CD | | Paed CD rank | Paed CD Δβ | Paed Holm adj P Value |
|---|---|---|---|---|---|---|---|---|---|---|---|---|
| 1 | 1 | cg17501210 | chr6 | RPS6KA2 | -0.07 | 3.08E-13 | -0.09 | 3.82E-19 | | 2 | -0.11 | 1.0E-09 |
| 2 | 2 | cg18608055 | chr19 | SBNO2 | -0.07 | 4.52E-13 | -0.08 | 1.00E-16 | | 29 | -0.14 | 7.5E-05 |
| 3 | 6 | cg09349128 | chr22 | NA | -0.04 | 1.19E-11 | -0.05 | 7.82E-13 | | 20 | -0.06 | 6.3E-05 |
| 4 | 3 | cg12170787 | chr19 | SBNO2 | -0.04 | 1.19E-11 | -0.05 | 2.91E-14 | | 39 | -0.11 | 1.9E-04 |
| 5 | 5 | cg16936953 | chr17 | VMP1 | -0.08 | 2.15E-11 | -0.10 | 1.19E-13 | | 4 | -0.16 | 3.0E-08 |
| 6 | 7 | cg12992827 | chr3 | NA | -0.05 | 4.15E-11 | -0.06 | 7.74E-11 | | 3 | -0.10 | 2.4E-08 |
| 7 | 12 | cg25114611 | chr6 | NA | -0.04 | 4.64E-11 | -0.04 | 6.84E-09 | | 102 | -0.04 | 1.3E-03 |
| 8 | 10 | cg02448796 | chr1 | KCNAB2 | 0.05 | 3.02E-10 | 0.05 | 2.98E-09 | | 2005 | 0.05 | 7.7E-02 |
| 9 | 4 | cg12054453 | chr17 | VMP1 | -0.07 | 7.25E-10 | -0.09 | 1.15E-13 | | 1 | -0.13 | 8.9E-10 |
| 10 | 21 | cg07398517 | chr3 | NA | -0.04 | 8.21E-10 | -0.04 | 3.40E-08 | | 27 | -0.06 | 7.4E-05 |
| 11 | 19 | cg13619623 | chr7 | BBS9 | 0.04 | 1.07E-09 | 0.04 | 2.31E-08 | | 13925 | 0.03 | 3.2E-01 |
| 12 | 65 | cg16724148 | chr1 | AGL | 0.03 | 2.70E-09 | 0.03 | 1.47E-06 | | 45887 | 0.01 | 5.2E-01 |
| 13 | 16 | cg26804423 | chr7 | ICA1 | 0.04 | 3.14E-09 | 0.04 | 1.25E-08 | | 13175 | 0.03 | 3.1E-01 |
| 14 | 17 | cg19821297 | chr19 | NA | -0.05 | 7.26E-09 | -0.05 | 1.83E-08 | | 9 | -0.08 | 9.2E-06 |
| 15 | 60 | cg22959742 | chr10 | FRMD4A | 0.05 | 7.26E-09 | 0.04 | 1.08E-06 | | 6879 | 0.04 | 2.1E-01 |
| 16 | 13 | cg03546163 | chr6 | FKBP5 | -0.06 | 7.96E-09 | -0.07 | 6.84E-09 | | 59 | -0.08 | 6.0E-04 |
| 17 | 64 | cg01059398 | chr3 | TNFSF10 | -0.04 | 1.31E-08 | -0.04 | 1.44E-06 | | 12 | -0.07 | 1.2E-05 |
| 18 | 25 | cg26955383 | chr10 | CALHM1 | 0.03 | 1.31E-08 | 0.04 | 4.04E-08 | | 38423 | 0.01 | 4.9E-01 |
| 19 | 32 | cg10636246 | chr1 | AIM2 | -0.04 | 1.74E-08 | -0.04 | 1.41E-07 | | 1736 | -0.05 | 6.7E-02 |
| 20 | 24 | cg18942579 | chr17 | VMP1 | -0.05 | 2.14E-08 | -0.05 | 4.04E-08 | | 15 | -0.10 | 2.5E-05 |
| 21 | 30 | cg01101459 | chr1 | NA | 0.04 | 3.15E-08 | 0.04 | 1.04E-07 | | 1145 | 0.06 | 4.3E-02 |
| 22 | 48 | cg17953136 | chr2 | LAPTM4A | 0.04 | 4.37E-08 | 0.04 | 4.84E-07 | | 24081 | 0.03 | 4.1E-01 |
| 23 | 38 | cg26020069 | chr6 | TRAM2 | -0.03 | 6.43E-08 | -0.03 | 2.13E-07 | | 1683 | -0.03 | 6.5E-02 |
| 24 | 15 | cg02716826 | chr9 | NA | -0.04 | 6.54E-08 | -0.04 | 1.07E-08 | | 17 | -0.06 | 6.3E-05 |
| 25 | 8 | cg25132241 | chr14 | FBLN5 | 0.02 | 6.54E-08 | 0.03 | 1.64E-10 | | 2545 | 0.03 | 9.9E-02 |
| 26 | 247 | cg27087650 | chr19 | BCL3 | -0.03 | 6.85E-08 | -0.02 | 0.000106 | | 5666 | -0.02 | 1.8E-01 |
| 27 | 54 | cg23172671 | chr1 | NA | 0.03 | 8.25E-08 | 0.03 | 7.99E-07 | | 5548 | 0.03 | 1.8E-01 |
| 28 | 72 | cg26470501 | chr19 | BCL3 | -0.03 | 8.29E-08 | -0.03 | 1.80E-06 | | 114 | -0.04 | 1.6E-03 |
| 29 | 26 | cg12670943 | chr22 | POLDIP3 | 0.02 | 8.29E-08 | 0.02 | 4.80E-08 | | 20023 | 0.02 | 3.8E-01 |
| 30 | 75 | cg07035242 | chr1 | UBIAD1 | 0.03 | 9.22E-08 | 0.03 | 2.01E-06 | | 65975 | 0.02 | 5.8E-01 |

*Supplementary Table 13 - Table comparing top DMPs in present adult 450k dataset (yellow, left of figure) with same probes in previously published paediatric Crohn's disease data from Adams et al (orange, right of figure). The hatched areas related to results for Crohn’s disease versus controls. Paed = Paediatric, adj = adjusted, CD = Crohn’s disease, IBD – Inflammatory bowel disease*

| Illumina 450k Probe id | Chr | Gene Symbol | Δβ | P.Value | FDR. adj.P.Val |
|---|---|---|---|---|---|
| cg24854010 | chr12 | WNT5B | 0.04 | 3.22E-10 | 0.0001 |
| cg03411579 | chr12 | NA | 0.06 | 2.02E-08 | 0.002 |
| cg00709979 | chr16 | NA | 0.04 | 2.61E-08 | 0.002 |
| cg17990365 | chr11 | IFITM3 | -0.09 | 3.02E-08 | 0.002 |
| cg18214661 | chr8 | PDGFRL | -0.05 | 3.91E-08 | 0.002 |
| cg23755933 | chr12 | GOLGA3 | 0.17 | 3.98E-08 | 0.002 |
| cg02508743 | chr8 | LYN | 0.08 | 4.22E-08 | 0.002 |
| cg19849557 | chr6 | C6orf136 | 0.08 | 4.63E-08 | 0.002 |
| cg19590591 | chr12 | GOLGA3 | 0.10 | 4.88E-08 | 0.002 |
| cg02143778 | chr16 | NA | 0.04 | 5.45E-08 | 0.002 |
| cg19045191 | chr11 | NAT10 | 0.05 | 6.87E-08 | 0.003 |
| cg01668008 | chr8 | NA | 0.03 | 1.25E-07 | 0.004 |
| cg06730953 | chr3 | NA | 0.03 | 1.36E-07 | 0.004 |
| cg24773560 | chr12 | IL23A | 0.05 | 1.47E-07 | 0.004 |
| cg13067095 | chr11 | EHD1 | 0.04 | 1.80E-07 | 0.004 |
| cg18959422 | chr1 | MYBPH | 0.05 | 1.98E-07 | 0.004 |
| cg06715330 | chr17 | CCDC57 | 0.05 | 2.02E-07 | 0.004 |

*Supplementary Table 14- Top Table of differentially methylated positions (DMPs) in inflammatory bowel disease (IBD) cases and controls in CD14+ monocytes.*

| Illumina 450k Probe id | Chr | Gene Symbol | Δβ | P.Value | FDR. adj.P.Val |
|---|---|---|---|---|---|
| cg09977980 | chr17 | EVPLL | 0.02 | 3.70E-08 | 0.006 |
| cg02498382 | chr2 | SNRNP27 | 0.02 | 3.97E-08 | 0.006 |
| cg22268279 | chr12 | FLJ12825 | 0.06 | 6.21E-08 | 0.006 |
| cg11823551 | chr5 | NA | 0.01 | 7.06E-08 | 0.006 |
| cg17572191 | chr11 | HRAS | 0.02 | 7.43E-08 | 0.006 |
| cg02478369 | chr17 | NA | 0.01 | 7.43E-08 | 0.006 |
| cg04587829 | chr17 | FN3K | 0.05 | 1.97E-07 | 0.010 |
| cg26481249 | chr4 | SORBS2 | 0.01 | 2.04E-07 | 0.010 |
| cg14031265 | chr4 | NA | 0.01 | 2.28E-07 | 0.010 |
| cg05195566 | chr15 | ISLR | 0.02 | 2.40E-07 | 0.010 |
| cg18608055 | chr19 | SBNO2 | -0.07 | 2.43E-07 | 0.010 |
| cg16123202 | chr7 | NA | 0.03 | 2.63E-07 | 0.010 |
| cg08835956 | chr7 | POU6F2 | 0.07 | 3.10E-07 | 0.011 |
| cg01243924 | chr1 | CNTN2 | 0.02 | 3.35E-07 | 0.011 |
| cg14811319 | chr6 | SPACA1 | 0.05 | 3.63E-07 | 0.011 |
| cg02465427 | chr22 | GUCD1 | 0.01 | 4.36E-07 | 0.011 |
| cg22617703 | chr20 | DNAJC5 | 0.02 | 4.56E-07 | 0.011 |

*Supplementary Table 15- Top Table of differentially methylated positions (DMPs) in inflammatory bowel disease (IBD) cases and controls in CD4+ lymphocytes. NA denotes methylation probes with no annotated gene symbol.*

| Illumina 450k Probe id | Chr | Gene Symbol | Δβ | P.Value | FDR. adj.P.Val |
|---|---|---|---|---|---|
| cg02985240 | chr1 | ARID4B | 0.07 | 1.34E-07 | 0.059 |
| cg27299024 | chr15 | NA | -0.02 | 3.89E-07 | 0.087 |
| cg07395000 | chr7 | TECPR1 | -0.01 | 7.71E-07 | 0.114 |
| cg11093149 | chr8 | UNC5D | -0.03 | 2.28E-06 | 0.209 |
| cg15390816 | chr12 | NCOR2 | 0.01 | 2.35E-06 | 0.209 |
| cg26949055 | chr15 | RYR3 | -0.01 | 3.28E-06 | 0.210 |
| cg26448609 | chr7 | LAMB1 | -0.02 | 4.73E-06 | 0.210 |
| cg07827038 | chr13 | NA | -0.01 | 5.28E-06 | 0.210 |
| cg25496181 | chr12 | KSR2 | 0.02 | 5.48E-06 | 0.210 |
| cg21069500 | chr5 | CXXC5 | 0.07 | 5.60E-06 | 0.210 |
| cg01454538 | chr2 | PXDN | 0.01 | 5.97E-06 | 0.210 |
| cg00505381 | chr2 | CAPN10 | 0.01 | 6.72E-06 | 0.210 |
| cg14189551 | chr2 | NA | 0.01 | 7.13E-06 | 0.210 |
| cg15093556 | chr7 | POLD2 | 0.01 | 7.14E-06 | 0.210 |
| cg21356040 | chr6 | MOG | 0.02 | 7.30E-06 | 0.210 |
| cg22708961 | chr1 | MORN1 | -0.02 | 7.53E-06 | 0.210 |
| cg05324393 | chr6 | NA | 0.06 | 8.94E-06 | 0.234 |

*Supplementary Table 16- Top Table of differentially methylated positions (DMPs) in inflammatory bowel disease (IBD) cases and controls in CD8+ lymphocytes. NA denotes methylation probes with no annotated gene symbol.*

| Illumina 450k Probe id | Chr | Gene Symbol | Δβ | P.Value | FDR. adj.P.Val |
|---|---|---|---|---|---|
| cg24854010 | chr12 | WNT5B | 0.05 | 6.65E-11 | 2.98E-05 |
| cg19515108 | chr12 | NA | 0.05 | 3.94E-09 | 0.0009 |
| cg01029592 | chr17 | SOX15 | 0.03 | 9.87E-09 | 0.001 |
| cg21408624 | chr7 | GRB10 | 0.01 | 1.43E-08 | 0.001 |
| cg18959422 | chr1 | MYBPH | 0.05 | 1.45E-08 | 0.001 |
| cg07532744 | chr12 | MON2 | -0.02 | 2.33E-08 | 0.002 |
| cg22017309 | chr11 | RAB3IL1 | -0.05 | 3.84E-08 | 0.002 |
| cg24127866 | chr17 | ABCA5 | -0.01 | 6.32E-08 | 0.003 |
| cg01368219 | chr3 | CACNA2D3 | 0.07 | 6.64E-08 | 0.003 |
| cg18470101 | chr4 | NA | 0.05 | 6.78E-08 | 0.003 |
| cg06179971 | chr17 | TBC1D16 | 0.02 | 8.05E-08 | 0.003 |
| cg15603957 | chr4 | CCSER1 | -0.03 | 8.59E-08 | 0.003 |
| cg19786988 | chr15 | TLN2 | 0.02 | 8.69E-08 | 0.003 |
| cg24521869 | chr17 | ABI3 | 0.06 | 1.56E-07 | 0.005 |
| cg01943221 | chr1 | PIK3CD | 0.03 | 2.09E-07 | 0.006 |
| cg22856496 | chr12 | ITGA7 | 0.07 | 2.19E-07 | 0.006 |
| cg10985810 | chr7 | PTPRN2 | 0.03 | 2.21E-07 | 0.006 |

*Supplementary Table 17 - Top table of differentially methylated positions (DMPs) between Crohn's disease (CD) cases and controls in CD14+ monocytes*

| Illumina 450k Probe id | Chr | Gene Symbol | Δβ | P.Value | FDR. adj.P.Val |
|---|---|---|---|---|---|
| cg20219381 | chr8 | RGS22 | 0.04 | 1.52E-07 | 0.0369 |
| cg11467680 | chr2 | NA | 0.02 | 1.64E-07 | 0.0369 |
| cg00345704 | chr17 | KRTAP2-3 | 0.01 | 2.59E-07 | 0.0387 |
| cg01243924 | chr1 | CNTN2 | 0.03 | 4.76E-07 | 0.0422 |
| cg26089160 | chr10 | PSD | 0.03 | 5.58E-07 | 0.0422 |
| cg04587829 | chr17 | FN3K | 0.06 | 7.55E-07 | 0.0422 |
| cg18413240 | chr3 | NA | -0.01 | 7.80E-07 | 0.0422 |
| cg00618291 | chr1 | SKI | 0.02 | 8.82E-07 | 0.0422 |
| cg09866283 | chr14 | NA | 0.01 | 9.19E-07 | 0.0422 |
| cg08503808 | chr8 | TRAPPC9 | 0.03 | 9.79E-07 | 0.0422 |
| cg14053997 | chr9 | ASS1 | 0.04 | 1.06E-06 | 0.0422 |
| cg02769267 | chr7 | MAD1L1 | 0.02 | 1.13E-06 | 0.0422 |
| cg18560366 | chr3 | DPPA4 | -0.04 | 1.68E-06 | 0.0478 |
| cg16051685 | chr1 | TRIM63 | 0.04 | 1.83E-06 | 0.0478 |
| cg15287594 | chr20 | FITM2 | -0.03 | 1.85E-06 | 0.0478 |
| cg00466500 | chr20 | CDH22 | 0.03 | 2.29E-06 | 0.0478 |
| cg10721834 | chr19 | NA | 0.06 | 2.38E-06 | 0.0478 |

*Supplementary Table 18- Top table of differentially methylated positions (DMPs) between Crohn's disease (CD) cases and controls in CD4+ lymphocytes*

| Illumina 450k Probe id | Chr | Gene Symbol | Δβ | P.Value | FDR. adj.P.Val |
|---|---|---|---|---|---|
| cg08113002 | chr19 | ASPDH | -0.09 | 1.40E-08 | 0.01 |
| cg08122156 | chr11 | SYT9 | 0.07 | 1.32E-06 | 0.15 |
| cg26716902 | chr17 | CNTNAP1 | 0.10 | 1.66E-06 | 0.15 |
| cg17296166 | chr19 | PNMAL1 | 0.04 | 1.90E-06 | 0.15 |
| cg22428858 | chr1 | NA | -0.01 | 2.41E-06 | 0.15 |
| cg20947775 | chr4 | SCD5 | 0.01 | 2.61E-06 | 0.15 |
| cg09789252 | chr19 | ASPDH | -0.08 | 2.77E-06 | 0.15 |
| cg11870206 | chr19 | ASPDH | -0.08 | 2.81E-06 | 0.15 |
| cg10702818 | chr3 | NA | 0.05 | 3.08E-06 | 0.15 |
| cg07370771 | chr10 | VENTX | 0.05 | 4.50E-06 | 0.19 |
| cg16575998 | chr2 | C2orf61 | 0.07 | 5.07E-06 | 0.19 |
| cg09794923 | chr2 | GPC1 | 0.02 | 5.59E-06 | 0.19 |
| cg13830646 | chr11 | FOSL1 | 0.01 | 5.69E-06 | 0.19 |
| cg02985240 | chr1 | ARID4B | 0.07 | 5.91E-06 | 0.19 |
| cg16429080 | chr2 | EXOC6B | -0.02 | 6.32E-06 | 0.19 |
| cg14178705 | chr6 | SKIV2L | -0.02 | 6.86E-06 | 0.19 |
| cg20573616 | chr10 | MMRN2 | -0.02 | 7.86E-06 | 0.21 |

*Supplementary Table 19 - Top table of differentially methylated positions (DMPs) between Crohn's disease (CD) cases and controls in CD8+ lymphocytes*

| Illumina 450k Probe id | Chr | Gene Symbol | Δβ | P.Value | FDR. adj.P.Val |
|---|---|---|---|---|---|
| cg08208133 | chr11 | HTR3A | 0.09 | 1.67E-09 | 0.0007 |
| cg08272368 | chr3 | NA | -0.04 | 9.74E-08 | 0.013 |
| cg11277662 | chr8 | TSNARE1 | -0.09 | 9.75E-08 | 0.013 |
| cg07665063 | chr12 | NA | 0.07 | 1.14E-07 | 0.013 |
| cg02508743 | chr8 | LYN | 0.10 | 2.02E-07 | 0.017 |
| cg03411579 | chr12 | NA | 0.07 | 2.54E-07 | 0.017 |
| cg01799015 | chr19 | PALM | -0.08 | 2.62E-07 | 0.017 |
| cg02230964 | chr8 | TSNARE1 | -0.09 | 3.07E-07 | 0.017 |
| cg23004985 | chr15 | PDE8A | -0.01 | 3.33E-07 | 0.017 |
| cg24985772 | chr11 | NA | 0.11 | 4.54E-07 | 0.017 |
| cg07842062 | chr16 | MEFV | 0.04 | 4.97E-07 | 0.017 |
| cg13300202 | chr8 | TSNARE1 | -0.05 | 5.43E-07 | 0.017 |
| cg26663590 | chr16 | NA | 0.10 | 5.50E-07 | 0.017 |
| cg04119529 | chr16 | NTHL1 | 0.04 | 5.77E-07 | 0.017 |
| cg12411704 | chr7 | ELFN1 | 0.06 | 5.77E-07 | 0.017 |
| cg02023138 | chr12 | BIN2 | 0.02 | 7.21E-07 | 0.019 |
| cg09678340 | chr16 | CMIP | 0.03 | 7.21E-07 | 0.019 |
| cg25189873 | chr13 | SERPINE3 | 0.03 | 7.97E-07 | 0.020 |
| cg25653947 | chr8 | NA | 0.05 | 8.84E-07 | 0.020 |
| cg08208133 | chr11 | HTR3A | 0.09 | 1.67E-09 | 0.0007 |

*Supplementary Table 20 - Top table of differentially methylated positions (DMPs) between Ulcerative colitis (UC) cases and controls in CD14+ Monocytes*

| Illumina 450k Probe id | Chr | Gene Symbol | Δβ | P.Value | FDR. adj.P.Val |
|---|---|---|---|---|---|
| cg02498382 | chr2 | SNRNP27 | 0.02 | 7.95E-09 | 0.004 |
| cg09977980 | chr17 | EVPLL | 0.02 | 4.60E-08 | 0.005 |
| cg05654304 | chr1 | VPS13D | 0.03 | 5.21E-08 | 0.005 |
| cg22268279 | chr12 | FLJ12825 | 0.08 | 5.39E-08 | 0.005 |
| cg18074189 | chr12 | TMTC1 | -0.06 | 6.09E-08 | 0.005 |
| cg23753748 | chr10 | CALHM2 | 0.06 | 8.68E-08 | 0.005 |
| cg18803306 | chr20 | NA | 0.07 | 1.10E-07 | 0.005 |
| cg19063856 | chr17 | RAPGEFL1 | -0.05 | 1.16E-07 | 0.005 |
| cg03638432 | chr16 | NA | -0.02 | 1.16E-07 | 0.005 |
| cg16677399 | chr6 | NA | -0.19 | 1.16E-07 | 0.005 |
| cg02886589 | chr2 | TMEM178A | -0.04 | 1.26E-07 | 0.005 |
| cg22217176 | chr18 | ATP9B | 0.04 | 1.56E-07 | 0.005 |
| cg11670802 | chr10 | DIP2C | 0.01 | 1.68E-07 | 0.005 |
| cg10359807 | chr16 | USP7 | -0.06 | 1.74E-07 | 0.005 |
| cg15986326 | chr10 | EPC1 | -0.06 | 1.82E-07 | 0.005 |
| cg22566355 | chr12 | KCNH3 | 0.04 | 2.09E-07 | 0.006 |
| cg26120842 | chr10 | NA | 0.04 | 2.75E-07 | 0.006 |
| cg09621572 | chr6 | LTA | -0.05 | 2.76E-07 | 0.006 |
| cg01620082 | chr3 | NA | -0.08 | 2.77E-07 | 0.006 |
| cg02498382 | chr2 | SNRNP27 | 0.02 | 7.95E-09 | 0.004 |

*Supplementary Table 21 - Top table of differentially methylated positions (DMPs) between Ulcerative colitis (UC) cases and controls in CD4+ Lymphocytes*

| Illumina 450k Probe id | Chr | Gene Symbol | Δβ | P.Value | FDR. adj.P.Val |
|---|---|---|---|---|---|
| cg00904852 | chr10 | NA | -0.04 | 2.90E-08 | 0.013 |
| cg04444506 | chr17 | KIF2B | -0.03 | 2.49E-07 | 0.036 |
| cg03160145 | chr18 | RAX | 0.03 | 5.56E-07 | 0.036 |
| cg04254389 | chr19 | NA | -0.07 | 7.94E-07 | 0.036 |
| cg05438837 | chr13 | CRYL1 | -0.02 | 8.09E-07 | 0.036 |
| cg12977937 | chr17 | NA | -0.06 | 8.15E-07 | 0.036 |
| cg01362776 | chr3 | NA | -0.02 | 8.86E-07 | 0.036 |
| cg19925178 | chr5 | NA | -0.04 | 9.46E-07 | 0.036 |
| cg08639523 | chr1 | NA | -0.03 | 1.08E-06 | 0.036 |
| cg22821931 | chr7 | GRB10 | -0.02 | 1.11E-06 | 0.036 |
| cg21543123 | chr14 | NA | -0.04 | 1.35E-06 | 0.036 |
| cg09852744 | chr6 | TULP4 | -0.07 | 1.39E-06 | 0.036 |
| cg04351185 | chr7 | NA | -0.04 | 1.43E-06 | 0.036 |
| cg08763102 | chr4 | HTT | -0.08 | 1.51E-06 | 0.036 |
| cg16624521 | chr5 | NA | 0.04 | 1.57E-06 | 0.036 |
| cg02985240 | chr1 | ARID4B | 0.08 | 1.59E-06 | 0.036 |
| cg25117822 | chr6 | NA | -0.07 | 1.61E-06 | 0.036 |
| cg03791799 | chr6 | MDC1 | -0.02 | 1.65E-06 | 0.036 |
| cg04898195 | chr1 | NPHP4 | 0.02 | 1.68E-06 | 0.036 |
| cg00904852 | chr10 | NA | -0.04 | 2.90E-08 | 0.013 |

*Supplementary Table 22 – Top table of differentially methylated positions (DMPs) between Ulcerative colitis (UC) cases and controls in CD8+ Lymphocytes*

| | CD | UC | IBD | Control | IBD versus Control |
|---|---|---|---|---|---|
| n | 22 | 22 | 44 | 24 | |
| Females (%) | 10 (45.5) | 10 (45.5) | 20 (45.5) | 8 (33) | 0.5 |
| Age At Diagnosis (median, IQR) | 26 (22-32) | 36.5 (26-50) | 28.2 (24.8-38.8) | 31.5 (25.7 – 41.3) | 0.7 |
| Smoking (current or Ex) | 13 (59) | 12 (57) | 25 (58.1) | 7 (32) | 0.08 |

*Supplementary Table 23 - Patient demographics of patients included in whole blood gene expression microarray (p values denote Fishers exact test for categorical variables, and Wilcoxon test for continuous variable)*

| | EntrezID (Seed) | Symbol (Seed) | Number of genes in network | Modularity | FDR p Value |
|---|---|---|---|---|---|
| 1 | 56616 | DIABLO | 30 | 3.628882 | 0.009 |
| 2 | 80331 | DNAJC5 | 38 | 2.701339 | 0.043 |
| 3 | 5265 | SERPINA1 | 14 | 4.457891 | 0.006 |
| 4 | 1991 | ELANE | 59 | 4.583397 | 0 |
| 5 | 4353 | MPO | 66 | 3.873013 | 0 |
| 6 | 3082 | HGF | 12 | 5.652254 | 0.002 |
| 7 | 566 | AZU1 | 13 | 4.021772 | 0.016 |
| 8 | 3674 | ITGA2B | 17 | 4.495567 | 0.004 |
| 9 | 1053 | CEBPE | 12 | 3.682455 | 0.032 |
| 10 | 966 | CD59 | 46 | 2.791665 | 0.026 |
| 11 | 4318 | MMP9 | 17 | 5.140515 | 0.002 |

*Supplementary Table 24- Functional epigenetic module for IBD versus control in whole blood. Modularity=average of edge weights. P Values are calculated using the Monte-Carlo procedure (a permutation test, n=1000)*

| | **Group 1** (low risk) (n=62) | **Group 2** (High risk) (n=91) | Wilcox p value (high risk versus low risk) | **Group 3** (intermediate risk) (n=87) | Kruskal-Wallis p value (all three groups) |
|---|---|---|---|---|---|
| **Age** | 37.6 (27-50.3) | 30.8 (24.6-47.7) | 0.1 | 33.4 (25-47.4) | 0.3 |
| **Female** (%) | 31 (50) | 36 (39.6) | 0.3 | 42 (48.3) | 0.4* |
| **Follow up length** | 18 (13-28) | 19 (7-25) | 0.6 | 20 (10-29) | 0.4 |
| **CRP** | 3.5 (1.25-5.75) | 14.5 (4-37.5) | 0.002 | 11 (2.25-26) | 0.004 |
| **Albumin** | 40 (37.25-41) | 34 (29-36.75) | 1.7E-5 | 37 (33-39) | 1.89E-5 |
| **Haemoglobin** | 141 (127-149.5) | 132.5 (115.5 – 142) | 0.02 | 130 (122-144) | 0.06 |

*Supplementary Table 25 - Demographics of three IBD subgroups generated by Unsupervised consensus clustering (data presented as medians (interquartile range), * = chi squared test)*

| | | |
|---|---|---|
| **ITGB2forward** | GGATAAAGAGGTGGGAATAGGTAAG | |
| **ITGB2reverse** | ATCCCTAATTAACAAAACCTCATCC | Biotin 5' |
| **ITGB2Sequencing** | GTATTGATATTTATTTGTATGTGA | |
| | | |
| **TXKforward** | ATAGGAAAATAGGTGGGGTTAA | |
| **TXKreverse** | CAAAAAAAAAAAATCATAATAACCCCTTCT | Biotin 5' |
| **TXKsequencing** | ATAGGTGGGGTTAATTT | |
| | | |
| **WRAP73forward** | TTTTTTTTTAGGGGTTTTTAGAAGGTG | |
| **WRAP73reverse** | TACCTAAACCAAATCCAACACATC | Biotin 5' |
| **WRAP73sequencing** | AGAAGGTGTTTAGATTTTAAG | |
| | | |
| **RPS6KA2forward** | GGTGGAGTTTATTGGAAGGTTGTG | |
| **RPS6KA2reverse** | ACAAAATCCCTCTAAATCCAACTATCT | Biotin 5' |
| **RPS6KA2sequencing** | TGGGTGGTTTATTTAGAAT | |
| | | |
| **SBNO2forward** | AGGAAAGAAGTTAGGGTTTGAT | Biotin 5' |
| **SBNO2reverse** | ACTCAATTACCCTCTCCTTTTTT | |
| **SBNO2sequencing** | CCTAAAAAACTAAATCACCAT | |
| | | |
| **VMP1forward** | TGGGTTATTGTATTTTGTTTTTAGTGTTG | |
| **VMP1reverse** | ACTAACAAACCAACTTCACTTATTTAC | Biotin 5' |
| **VMP1sequencing** | GTATTTTGTTTTTAGTGTTGTT | |

*Supplementary Table 26 - Pyrosequencing primer sequences*

## Supplementary References


1. Houseman, E. A. *et al.* DNA methylation arrays as surrogate measures of cell mixture distribution. *BMC Bioinformatics* **13,** 86 (2012).

2. Taylor, P. *et al.* Epigenetics Cigarette smoking reduces DNA methylation levels at multiple genomic loci but the effect is partially reversible upon cessation. *Epigenetics* **9,** 1382–1396 (2014).

3. Horvath, S. DNA methylation age of human tissues and cell types. *Genome Biol* **14,** R115 (2013).

4. Adams, A. T. *et al.* Two-stage genome-wide methylation profiling in childhood-onset Crohn's Disease implicates epigenetic alterations at the VMP1/MIR21 and HLA loci. *Inflamm. Bowel Dis.* **20,** 1784–93 (2014).

5. Lee, J. C. *et al.* Gene expression profiling of CD8+ T cells predicts prognosis in patients with Crohn disease and ulcerative colitis. *J. Clin. Invest.* **121,** 4170–9 (2011).

6. Calvo-Garrido, J., Carilla-Latorre, S. & Escalante, R. Vacuole membrane protein 1, autophagy and much more. *Autophagy* **4,** 835–7 (2008).

7. Molejon, M. I., Ropolo, A., Re, A. Lo, Boggio, V. & Vaccaro, M. I. The VMP1-Beclin 1 interaction regulates autophagy induction. *Sci. Rep.* **3,** 1055 (2013).

8. Kang, R., Zeh, H. J., Lotze, M. T. & Tang, D. The Beclin 1 network regulates autophagy and apoptosis. *Cell Death Differ.* **18,** 571–80 (2011).

9. Ribas, J. *et al.* A novel source for miR-21 expression through the alternative polyadenylation of VMP1 gene transcripts. *Nucleic Acids Res.* **40,** 6821–33 (2012).

10. Kanaan, Z. *et al.* Plasma miR-21: a potential diagnostic marker of colorectal cancer. *Ann. Surg.* **256,** 544–51 (2012).

11. Kalla, R. *et al.* MicroRNAs: new players in IBD. *Gut* 1–14 (2014). doi:10.1136/gutjnl-2014-307891

12. Shi, C. *et al.* MicroRNA-21 knockout improve the survival rate in DSS induced fatal colitis through protecting against inflammation and tissue injury. *PLoS One* **8,** e66814 (2013).

13. Wu, F. *et al.* Divergent influence of microRNA-21 deletion on murine colitis phenotypes. *Inflamm. Bowel Dis.* **20,** 1972–85 (2014).

14. Wu, F. *et al.* MicroRNAs are differentially expressed in ulcerative colitis and alter expression of macrophage inflammatory peptide-2 alpha. *Gastroenterology* **135,** 1624–

1635.e24 (2008).

15. Wu, F., Zhang, S., Dassopoulos, T. & Harris, M. L. Identification of MicroRNAs Associated with Ileal and Colonic Crohn's Disease. *Inflamm. Bowel Dis.* **16,** 1729–1738 (2010).

16. Takagi, T. *et al.* Increased expression of microRNA in the inflamed colonic mucosa of patients with active ulcerative colitis. *J. Gastroenterol. Hepatol.* **25 Suppl 1,** S129–33 (2010).

17. Fasseu, M. *et al.* Identification of restricted subsets of mature microRNA abnormally expressed in inactive colonic mucosa of patients with inflammatory bowel disease. *PLoS One* **5,** e13160 (2010).

18. Zahm, A. M. *et al.* Circulating MicroRNA is a biomarker of pediatric Crohn disease. *J. Pediatr. Gastroenterol. Nutr.* **53,** 26–33 (2011).

19. Koshizuka, Y., Ikegawa, S., Sano, M., Nakamura, K. & Nakamura, Y. Isolation, characterization, and mapping of the mouse and human WDR8 genes, members of a novel WD-repeat gene family. *Genomics* **72,** 252–259 (2001).

20. Tan, S. The leucocyte β2 (CD18) integrins: the structure, functional regulation and signalling properties. *Biosci. Rep.* **32,** 241–269 (2012).

21. Kishimoto, T. K., Hollander, N., Roberts, T. M., Anderson, D. C. & Springer, T. A. Heterogeneous mutations in the beta subunit common to the LFA-1, Mac-1, and p150,95 glycoproteins cause leukocyte adhesion deficiency. *Cell* **50,** 193–202 (1987).

22. van de Vijver, E., van den Berg, T. K. & Kuijpers, T. W. Leukocyte Adhesion Deficiencies. *Hematol. Oncol. Clin. North Am.* **27,** 101–116 (2013).

23. Harris, R. A. *et al.* DNA methylation-associated colonic mucosal immune and defense responses in treatment-naïve pediatric ulcerative colitis. *Epigenetics* **9,** 1131–7 (2014).

24. Hutterer, E. *et al.* CD18 (ITGB2) expression in chronic lymphocytic leukaemia is regulated by DNA methylation-dependent and -independent mechanisms. *Br. J. Haematol.* **169,** 286–9 (2015).

25. Stefano Nardone, Dev Sharan Sams, Eli Reuveni, Dmitriy Getselter, Oded Oron, Marcela Karpuj, E. E. DNA methylation analysis of the autistic brain reveals multiple dysregulated biological pathways. *Transl. Psychiatry* (2014). doi:10.1038/tp.2014.70

26. Langer-Gould, A., Atlas, S. W., Green, A. J., Bollen, A. W. & Pelletier, D. *Progressive multifocal leukoencephalopathy in a patient treated with natalizumab. N. Engl. J. Med.* **353,** 375–381 (2005).

27. Feagan, B. G. *et al.* Vedolizumab as induction and maintenance therapy for ulcerative colitis. *N. Engl. J. Med.* **369,** 699–710 (2013).

28. Sandborn, W. J. *et al.* Vedolizumab as induction and maintenance therapy for Crohn's disease. *N. Engl. J. Med.* **369,** 711–21 (2013).

29. Haire, R. N. *et al.* TXK, a novel human tyrosine kinase expressed in T cells shares sequence identity with Tec family kinases and maps to 4p12. *Hum. Mol. Genet.* **3,** 897–901 (1994).

30. Gomez-Rodriguez, J., Kraus, Z. J. & Schwartzberg, P. L. Tec family kinases Itk and Rlk/Txk in T lymphocytes: Cross-regulation of cytokine production and T-cell fates. *FEBS J.* **278,** 1980–1989 (2011).

31. Nagafuchi, H. *et al.* Excessive expression of Txk, a member of the Tec family of tyrosine kinases, contributes to excessive Th1 cytokine production by T lymphocytes in patients with Behcet's disease. *Clin. Exp. Immunol.* **139,** 363–370 (2005).

32. Suzuki, N., Nara, K. & Suzuki, T. Skewed Th1 responses caused by excessive expression of Txk, a member of the Tec family of tyrosine kinases, in patients with Behcet's disease. *Clin. Med. Res.* **4,** 147–151 (2006).

33. Jostins, L. *et al.* Host-microbe interactions have shaped the genetic architecture of inflammatory bowel disease. *Nature* **490,** 119–124 (2012).

34. Shahbazian, M. D. & Grunstein, M. Functions of site-specific histone acetylation and deacetylation. *Annu. Rev. Biochem.* **76,** 75–100 (2007).

35. Butzner, J. D., Parmar, R., Bell, C. J. & Dalal, V. Butyrate enema therapy stimulates mucosal repair in experimental colitis in the rat. *Gut* **38,** 568–73 (1996).

36. Rönn, T. *et al.* A Six Months Exercise Intervention Influences the Genome-wide DNA Methylation Pattern in Human Adipose Tissue. *PLoS Genet.* **9,** (2013).

37. Benton, M. C. *et al.* An analysis of DNA methylation in human adipose tissue reveals differential modification of obesity genes before and after gastric bypass and weight loss. *Genome Biol.* **16,** 8 (2015).

38. Maller, J. L., Foulkes, J. G., Erikson, E. & Baltimore, D. Phosphorylation of ribosomal protein S6 on serine after microinjection of the Abelson murine leukemia virus tyrosine-specific protein kinase into Xenopus oocytes. *Proc. Natl. Acad. Sci. U. S. A.* **82,** 272–276 (1985).

39. Anjum, R. & Blenis, J. The RSK family of kinases: emerging roles in cellular signalling. *Nat. Rev. Mol. Cell Biol.* **9,** 747–758 (2008).

40. Pancholi, S. *et al.* ERBB2 influences the subcellular localization of the estrogen receptor in

tamoxifen-resistant MCF-7 cells leading to the activation of AKT and RPS6KA2. *Endocr. Relat. Cancer* **15,** 985–1002 (2008).

41. Bignone, P. A. *et al.* RPS6KA2, a putative tumour suppressor gene at 6q27 in sporadic epithelial ovarian cancer. *Oncogene* **26,** 683–700 (2007).

42. El Kasmi, K. C. *et al.* Cutting Edge: A Transcriptional Repressor and Corepressor Induced by the STAT3-Regulated Anti-Inflammatory Signaling Pathway. *J. Immunol.* **179,** 7215–7219 (2007).

43. Franke, A. *et al.* Meta-Analysis Increases to 71 the Tally of Confirmed Crohn's Disease Susceptibility Loci. *Nat. Genet.* **42,** 1118–1125 (2010).

44. Wiley, S. R. *et al.* Identification and characterization of a new member of the TNF family that induces apoptosis. *Immunity* **3,** 673–682 (1995).

45. Almasan, A. & Ashkenazi, A. Apo2L/TRAIL: Apoptosis signaling, biology, and potential for cancer therapy. *Cytokine Growth Factor Rev.* **14,** 337–348 (2003).

46. Johnstone, R. W., Frew, A. J. & Smyth, M. J. The TRAIL apoptotic pathway in cancer onset, progression and therapy. *Nat. Rev. Cancer* **8,** 782–798 (2008).

47. Begue, B. *et al.* Implication of TNF-Related Apoptosis-Inducing Ligand in Inflammatory Intestinal Epithelial Lesions. *Gastroenterology* **130,** 1962–1974 (2006).

48. Brost, S. *et al.* Differential expression of the TRAIL/TRAIL-receptor system in patients with inflammatory bowel disease. *Pathol. Res. Pract.* **206,** 43–50 (2010).

49. McKeithan, T. W., Ohno, H., Dickstein, J. & Hume, E. Genomic structure of the candidate proto-oncogene BCL3. *Genomics* **24,** 120–126 (1994).

50. Anderson, C. A. *et al.* Meta-analysis identifies 29 additional ulcerative colitis risk loci, increasing the number of confirmed associations to 47. *Nat. Genet.* **43,** 246–252 (2011).

51. Brand, S. Crohn's disease: Th1, Th17 or both? The change of a paradigm: new immunological and genetic insights implicate Th17 cells in the pathogenesis of Crohn's disease. *Gut* **58,** 1152–67 (2009).
